# Rosetta:MSF: a modular framework for multi-state computational protein design

**DOI:** 10.1371/journal.pcbi.1005600

**Published:** 2017-06-12

**Authors:** Patrick Löffler, Samuel Schmitz, Enrico Hupfeld, Reinhard Sterner, Rainer Merkl

**Affiliations:** Institute of Biophysics and Physical Biochemistry, University of Regensburg, Regensburg, Germany; The Pennsylvania State University, UNITED STATES

## Abstract

Computational protein design (CPD) is a powerful technique to engineer existing proteins or to design novel ones that display desired properties. Rosetta is a software suite including algorithms for computational modeling and analysis of protein structures and offers many elaborate protocols created to solve highly specific tasks of protein engineering. Most of Rosetta’s protocols optimize sequences based on a single conformation (*i*. *e*. design state). However, challenging CPD objectives like multi-specificity design or the concurrent consideration of positive and negative design goals demand the simultaneous assessment of multiple states. This is why we have developed the multi-state framework MSF that facilitates the implementation of Rosetta’s single-state protocols in a multi-state environment and made available two frequently used protocols. Utilizing MSF, we demonstrated for one of these protocols that multi-state design yields a 15% higher performance than single-state design on a ligand-binding benchmark consisting of structural conformations. With this protocol, we designed *de novo* nine retro-aldolases on a conformational ensemble deduced from a (βα)_8_-barrel protein. All variants displayed measurable catalytic activity, testifying to a high success rate for this concept of multi-state enzyme design.

## Author summary

Protein engineering, *i*. *e*. the targeted modification or design of proteins has tremendous potential for medical and industrial applications. One generally applicable strategy for protein engineering is rational protein design: based on detailed knowledge of structure and function, computer programs like Rosetta propose the sequence of a protein possessing the desired properties. So far, most computer protocols have used rigid structures for design, which is a simplification because a protein’s structure is more accurately specified by a conformational ensemble. We have now implemented a framework for computational protein design that allows certain design protocols of Rosetta to make use of multiple design states like structural ensembles. An *in silico* assessment simulating ligand-binding design showed that this new approach generates more reliably native-like sequences than a single-state approach. As a proof-of-concept, we introduced *de novo* retro-aldolase activity into a scaffold protein and characterized nine variants experimentally, all of which were catalytically active.

## Introduction

Since the 1990s, computational protein design (CPD) has been a powerful tool of protein engineering. For example, CPD has been successfully utilized to increase thermostability of proteins [1–3] and to design new or altered binding specificities for metals [4], DNA [5] or other ligands [6, 7]. Additionally, CPD was applied to even more challenging tasks like the design of novel protein-protein interfaces [8, 9], *de novo* enzymes [10] or artificial folds not found in nature [11, 12]. Classical CPD methods, referred to as single-state design (SSD), optimize the amino acid sequence for the residue positions of a single backbone by means of an objective function [13]. A substantial contribution to the enormous success reached by SSD is due to refinements of the corresponding knowledge-based or statistical energy terms and the incorporation of backbone flexibility [14]. However, SSD is always a simplification because proteins populate conformational ensembles [15]. Moreover, certain design objectives such as negative design [16–18], multi-specificity design [19], the design of specific protein interfaces [20, 21] or the mimicking of backbone flexibility [22] require the concurrent assessment of several conformational or chemical states. This is why multi-state design (MSD) methodology is an emerging field in CPD [23] that extends the application spectrum and promises high success rates. Even the design of stable proteins profits from using backbone ensembles [24].

Typically, the optimization strategy of MSD consists of an “outer routine” that suggests possible amino acids sequences and an “inner routine” that assesses the fitness of a given sequence by performing rotamer optimization on each of the considered states and combines the individual scores [25]. This combined score enables a sequence selection driven by the energetic contribution of multiple conformational and/or chemical states. For example, in order to increase specificity of protein-protein interactions, one can utilize negative design and penalize those sequences that favor undesired interactions [16].

One of the first applications of MSD was the design of topologically specific coiled-coil structures consisting of 11-fold amino acid repeats whose stability was assessed by using terms of a standard molecular-mechanics potential energy function [26]. Later on, the binding pocket of a ribose-binding protein was successfully redesigned by means of MSD based on a standard force-field [27]. Meanwhile, many of the common optimization algorithms used in SSD have been adapted for MSD, including Monte Carlo (MC) with simulated annealing [28], genetic algorithms [29], the FASTER approach [25], dead-end-elimination [30], and cluster expansion [31]. Rosetta [32] is currently the most flexible and most widely used CPD software suite and offers several multi-state applications; noteworthy are MPI_MSD [33] and RECON [34]. MPI_MSD provides a generic multi-state design implementation based on a genetic algorithm that optimizes a single sequence on multiple states given a fitness function. RECON starts by individually optimizing one sequence for each state; subsequently the computation of a consensus sequence is promoted by incrementally increasing convergence restraints. However, the current implementations of both methods are limited to certain design tasks and cannot make use of fine-tuned protocols like those required for enzyme design [35] or anchored design of protein-protein interfaces [36].

In order to overcome this limitation, we have developed MSF and our integration of this modular framework into Rosetta facilitates the transfer of already proven single-state protocols to an MSD environment. Here, by using MSF, we first corroborate the superiority of MSD for enzyme design based on two *in silico* benchmarks for ligand binding. Applying the same protocol, we then designed nine experimentally active retro-aldolases.

## Results and discussion

### Architecture of MSF

**Fig 1:**
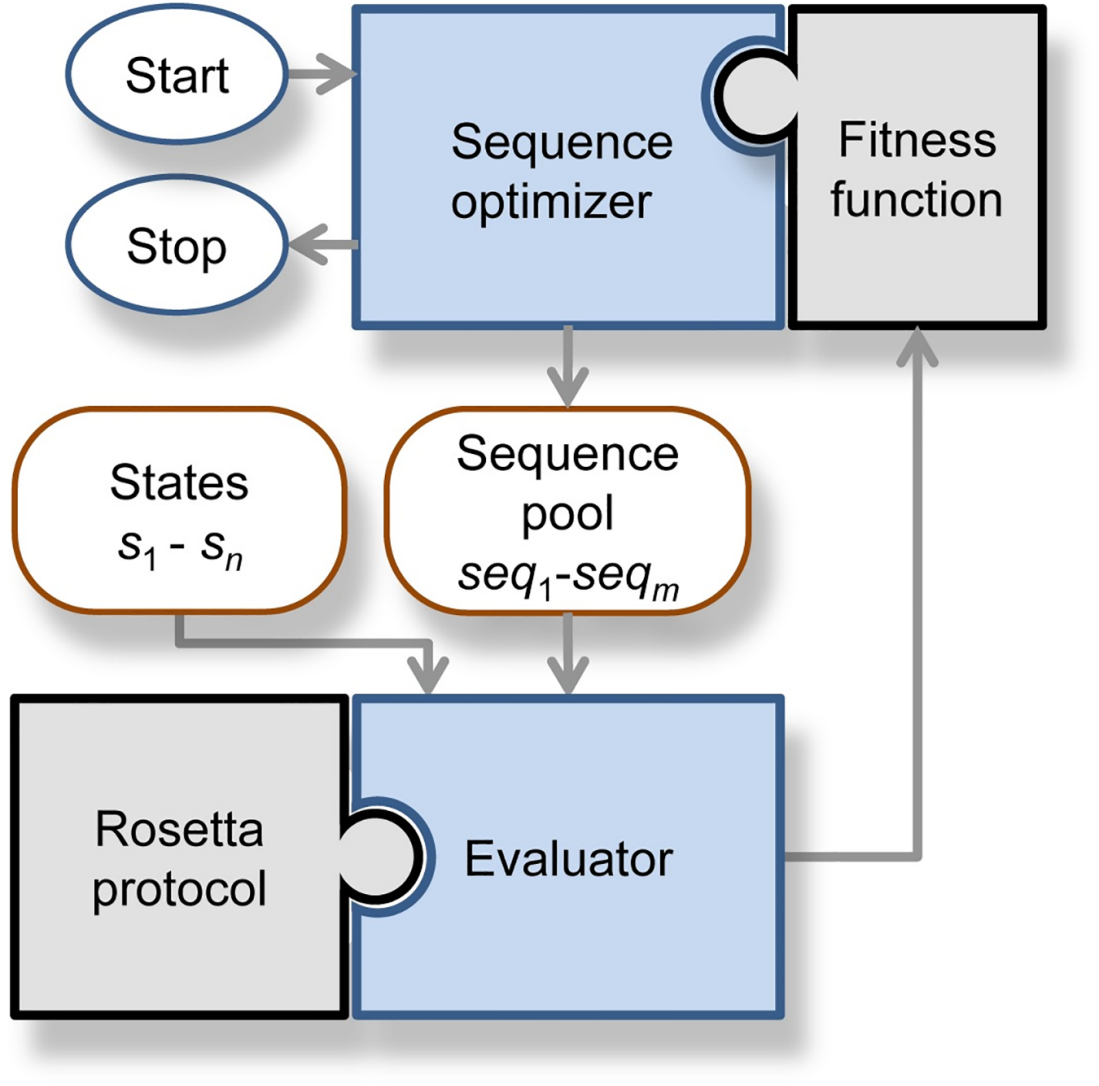
Software architecture of MSF. This framework consists of two strictly separated modules, the *sequence optimizer* and the *evaluator*. The *evaluator* executes the chosen *Rosetta protocol* for each combination of a state *s*_*j*_ and a sequence *seq*_*i*_. The resulting scores are processed by the *fitness function* and transferred to the *sequence optimizer*. Initially, the user has to specify a number of states *s*_1_, …, *s*_*n*_ and a set of initial sequences *seq*_1_,…, *seq*_*m*_. MSF uses a GA to optimize the sequences according to their fitness. To utilize a SSD protocol in an MSD environment, the user has to adapt the protocol to the *evaluator* and specify a *fitness function*.

MSF is a programming framework that allows the user to develop and execute Rosetta protocols in an MSD environment. The modular software architecture of MSF significantly reduces the development efforts involved; see Fig 1.

MSF requires as input a set of states *s*_1_, …, *s*_*n*_, e. g. structural conformations, and a population of sequences *seq*_1_, …, *seq*_*m*_, which will be subsequently altered by the *sequence optimizer*. The *evaluator* determines *n* state-specific scores for each *seq*_*i*_ according to the chosen *Rosetta protocol*. These *n* × *m* scores are the input of a user-defined *fitness function*, which combines the scores to determine the fitness of each sequence and communicates these values to the *sequence optimizer*. The task management is as follows for all protocols: one process controls the *sequence optimizer* and a user-defined number of *evaluator*-processes execute the protocol in parallel, which guarantees high scalability. Technical details and availability are described in S1 Text; MSF will be part of an upcoming weekly release of Rosetta.

As has been shown, a genetic algorithm (GA) successfully samples sequence space in MSD calculations [16, 27, 33]. Therefore, we have implemented the *sequence optimizer* based on the well-proven GA of Rosetta. Briefly, a GA imitates the process of natural selection by maintaining a population of design sequences that are evolving for a number of generations, while the selection pressure of the *fitness function* eliminates less optimal solutions. The final output of MSF is a population of optimized sequences. By contrast, a standard SSD implementation that utilizes MC optimization generates one sequence.

### Extending Rosetta protocols by MSD capability

Both MSF and MPI_MSD rely on the Rosetta GA. However, MPI_MSD does not support the integration of existing SSD protocols such as enzyme design that requires the additional optimization of catalytic constraints. Thus, our aim was to offer a framework that minimizes the development effort of supplying SSD protocols with MSD capability. The architecture of MSF strictly separates the tasks of optimization and the application-specific assessment of states. The resulting modularity allows an informed Rosetta user to implement MSD for existing protocols in a straightforward manner. Most importantly, the functionality of the protocols is unchanged and all options remain available. In addition to protocol porting, the user has to set up an application-specific *fitness function*, which defines the design goal.

If it is the goal to alter conformational, binding, or catalytic specificity, the *fitness function* often has to consider positive and negative design. For the assessment of one positive state *s*_*+*_ and one negative state *s*_*-*_, the following function has been proposed [25]:

$$fitness_{+,-}(seq_{i})=\Delta score_{+}(seq_{i})-w\;\Delta score_{-}(seq_{i})$$ (1)

Here, Δ*score*_*l*_ (*seq*_*i*_) is the difference of scores calculated for *seq*_*i*_ and *seq*_0_; *seq*_0_ is the optimal sequence determined in an SSD for the states *s*_*l*_ ∈ {*s*_+_,*s*_−_} and *w* is a weighting factor. Similar approaches, which were based on the computed transfer free energy from the target state to the ensemble of competing states [16] or on differences of Rosetta energies [33] guided the MSD of protein interfaces. Equally to MPI_MSD, our framework MSF supports the specification of a broad range of fitness functions.

For the initial implementation of MSF, we have integrated enzdes and AnchoredDesign, two widely used Rosetta protocols. enzdes provides ligand binding and enzyme design functionality by repacking and redesigning residues around the binding/active site and by optimizing catalytic contacts. AnchoredDesign creates a protein-interface by transferring a key interaction identified in a natural binding partner of the target protein to a surface loop of the scaffold protein. Afterwards, the surface of the scaffold is redesigned with backbone flexibility to generate a novel binding partner of the target [36].

To validate AnchoredDesign in the MSF context, we redesigned the interface of the factor B serine protease domain from *Homo sapiens* (PDB ID 1dle). For this single example, the MSD approach performed better that the corresponding SSD protocol; see S2 Text for details. In order to demonstrate the potential of MSF for a large number of cases, we focused on enzdes by performing *in silico* and *in vitro* experiments. For the *in silico* assessment, the fitness of the sequences was computed according to Eq 2 based on the Rosetta total score (*ts*) averaged over all states. In the following, we designate software protocols as program:protocol. For example, Rosetta:enzdes (or for the sake of brevity enzdes) and Rosetta:MSF:GA:enzdes (MSF:GA:enzdes) are the names of the SSD and MSD implementations of enzdes.

### MSD outperforms SSD in recapitulating a ligand binding site of an NMR ensemble

The most obvious usage of MSD is its application to an ensemble representing the native conformations of a protein. In solution, a protein’s structure is dynamic and nuclear magnetic resonance (NMR) offers an experimentally determined estimation of protein dynamics. Interestingly, in previous analyses SSD protocols performed better on crystal structures than on NMR templates [22, 37]. We speculated that this performance loss can be compensated, if MSD is applied to a whole ensemble and we decided to assess a ligand-binding design.

Thus, for a first performance comparison of the SSD algorithm enzdes, and the MSD algorithm MSF:GA:enzdes, we chose an NMR ensemble of the human intestinal fatty acid binding protein (hIFABP) with bound ketorolac (PDB ID 2mji). This ensemble consisting of ten conformations was prepared for ligand-binding design (see Materials and Methods) and the design shell contained 21 residue positions in the vicinity of the ligand. Our protocol allowed Rosetta to find a low energy sequence by arbitrarily choosing residues for these positions.

For each of the individual conformations *conf*(*l*), 1000 randomly seeded *runs*_*l*_ (*i*) of enzdes (SSD) were started. Design quality was monitored by computing for each number of runs *i* the score $ts{\;}_{SSD}^{hIFABP}(i)$. This is the mean total score deduced from corresponding conformations (Eq 6) given in Rosetta Energy Units (REU). MSF:GA:enzdes (MSD) was applied to the full ensemble and the GA was started. Analogously to the SSD experiment, the mean total score $ts{\;}_{MSD}^{hIFABP}(j)\;$ was computed for each generation *j* (Eq 7). As a second measure of design quality, we determined the native sequence similarity recovery (*nssr*). Commonly, the performance of design algorithms is assessed by means of the native sequence recovery (*nsr*) [38–40], which is the fraction of identical residues at corresponding positions of the native and the designed sequence. The concept of *nsr* is blind for a more specific comparison of residues beyond identity, which may impede a detailed assessment. In contrast, for the computation of *nssr*, all residue pairs reaching a BLOSUM62 score > 0 are considered similar and contribute to the *nssr* value (Eqs 4 and 5).

**Fig 2:**
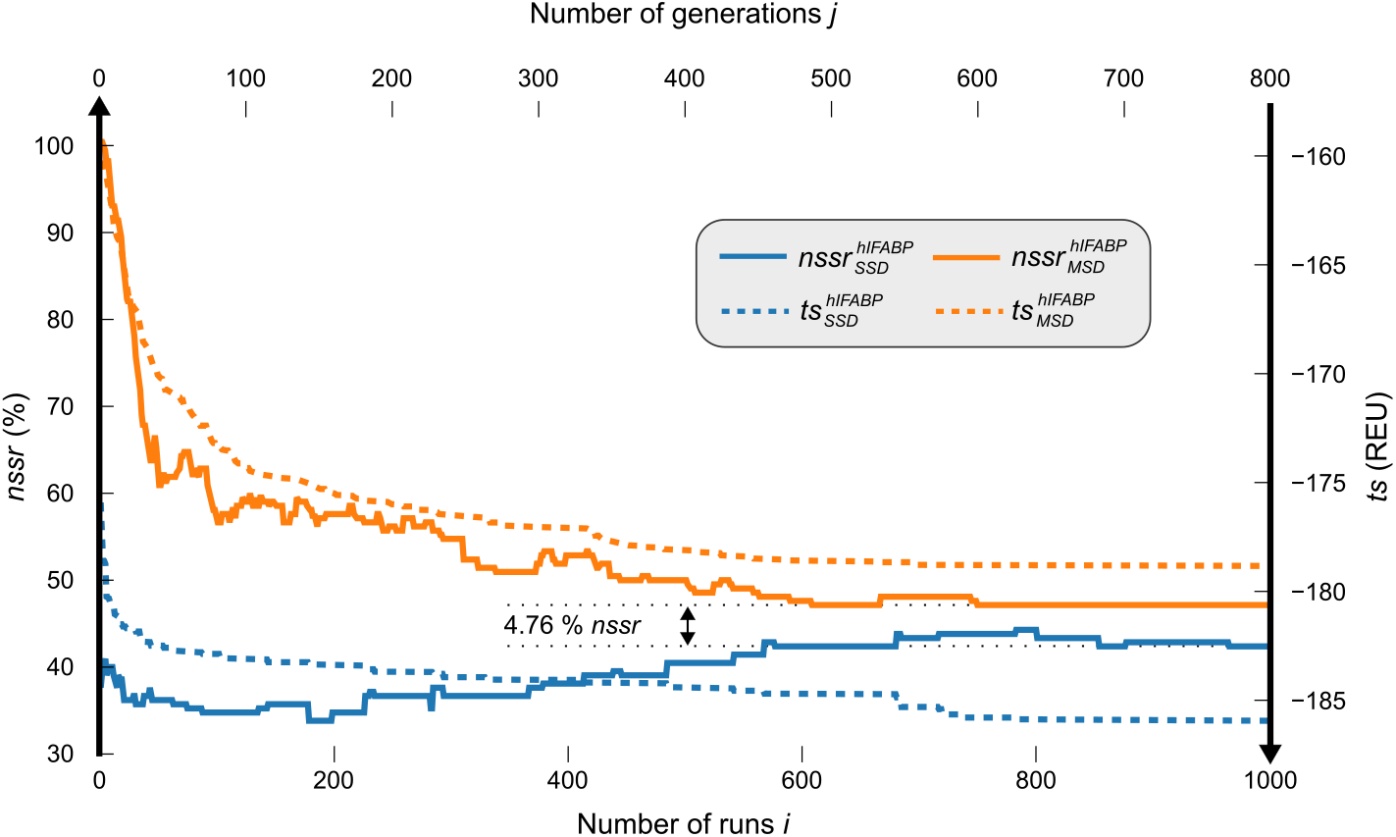
Performance of SSD and MSD on the NMR ensemble hIFABP. enzdes (blue lines) was executed for 1000 runs *i* for each of the ten conformations in the ensemble. For each number of runs *i*, the $ts{\;}_{SSD}^{hIFABP}(i)$ value (dotted line) is the mean of the ten lowest-energy sequences (Eq 6). The corresponding $nssr{\;}_{SSD}^{hIFABP}(i)$ value (solid line) is the mean recovery value deduced from the same sequences (Eq 5). MSF:GA:enzdes (orange lines) was carried out for 800 generations *j* on the whole ensemble using a population of 210 sequences. For each generation *j*, the $ts{\;}_{MSD}^{hIFABP}(j)$ value (dotted line) is the mean of the ten lowest-energy sequences of the corresponding population (Eq 7). The corresponding $nssr{\;}_{MSD}^{hIFABP}(j)$ value (solid line) is the mean recovery value deduced from the same sequences (Eq 5).

The plots shown in Fig 2 indicate that the SSD and the MSD algorithm converged after 1000 runs or 800 generations, respectively, both with respect to sequence recovery and *ts* values of the chosen sequences. The mean *nsr* values of enzdes and of MSF:GA:enzdes were 20.00% and 27.14%, and the mean *nssr* values were 41.90% and 46.66%. Only two of the ten enzdes designs reached an *nssr* value (47.62% and 61.90%, respectively) that was higher than the mean *nssr* of MSF:GA:enzdes. In summary, MSF:GA:enzdes performed better than enzdes suggesting the usage of MSD if sequences have to be designed for an ensemble.

Altogether, the energies of models generated in SSD were on average 7.11 REU lower than those in MSD. However, a comparison of *ts* scores is no ideal means to compare SSD and MSD performance. In MSD, a sequence is a compromise that has to satisfy the constraints associated with all conformations in an acceptable manner. In contrast, SSD customizes a low energy sequence for each conformation. Thus, it is no surprise that the mean *ts* values of SSD sequences are superior to those of the MSD results. On the other hand, due to these specific adaptations based on single, less-native conformations, the SSD sequences are receding from the native ones, which are considered as close to optimal [41]. This undesired effect is less pronounced for MSD sequences computed on the whole native ensemble. We conclude that *nsr* and *nssr* are more suitable than *ts* values for a comparative benchmarking of SSD and MSD approaches.

### A novel benchmark dataset for ligand-binding based on conformational sampling

A standard dataset for the assessment of ligand-binding and enzyme design is the *enzdes scientific sequence recovery benchmark*. It consists of 51 representative proteins in which the ligand is bound with an affinity of 10 μM or lower [42]. During benchmarking, a given CPD algorithm redesigns residues of the design shell enclosing each ligand and the algorithm’s ability to recapitulate the native sequence (*nsr* and *nssr* values) is measured. However, for an assessment of *de novo* design algorithms, this approach may be misleading, because the required remodeling of a chosen protein is more demanding than the recapitulation of its native binding pocket.

We created a more realistic benchmark that is devoid of a perfect backbone/rotamer preorganization and is more suitable for the assessment of *de novo* design algorithms. For feasibility reasons, we randomly selected 16 proteins *prot*(*k*) of the above 51 benchmark proteins. The corresponding ligands were removed and for each of the 16 apoproteins, an ensemble of 20 conformations was created using the Backrub server [43], which generates near-native conformational ensembles [44, 45]. Next, by superposition of each conformation with the corresponding crystal structure, the ligands were transferred to the binding pockets. Thus, the resulting dataset *BR_EnzBench* featured for each of the 16 *prot*(*k*) 20 backbone conformations that are near to native but lack the implicit pre-organization induced by a bound ligand in a crystal structure.

### MSD outperforms SSD on a benchmark dataset mimicking *de novo* design applications

We used *BR_EnzBench* to compare the performance of SSD and MSD for *de novo* ligand-binding design. All design shell residues were initially mutated to alanine and the conformations were energy-minimized to further increase the difficulty for CPD algorithms to recover the native sequence. To prevent a hydrophobic collapse of the alanine-only design shells, minimization was performed with backbone constraints. Thus, the CPD problem to be solved within the scope of this benchmark was to design a binding pocket by sequence optimization of the all-alanine design shells.

**Fig 3:**
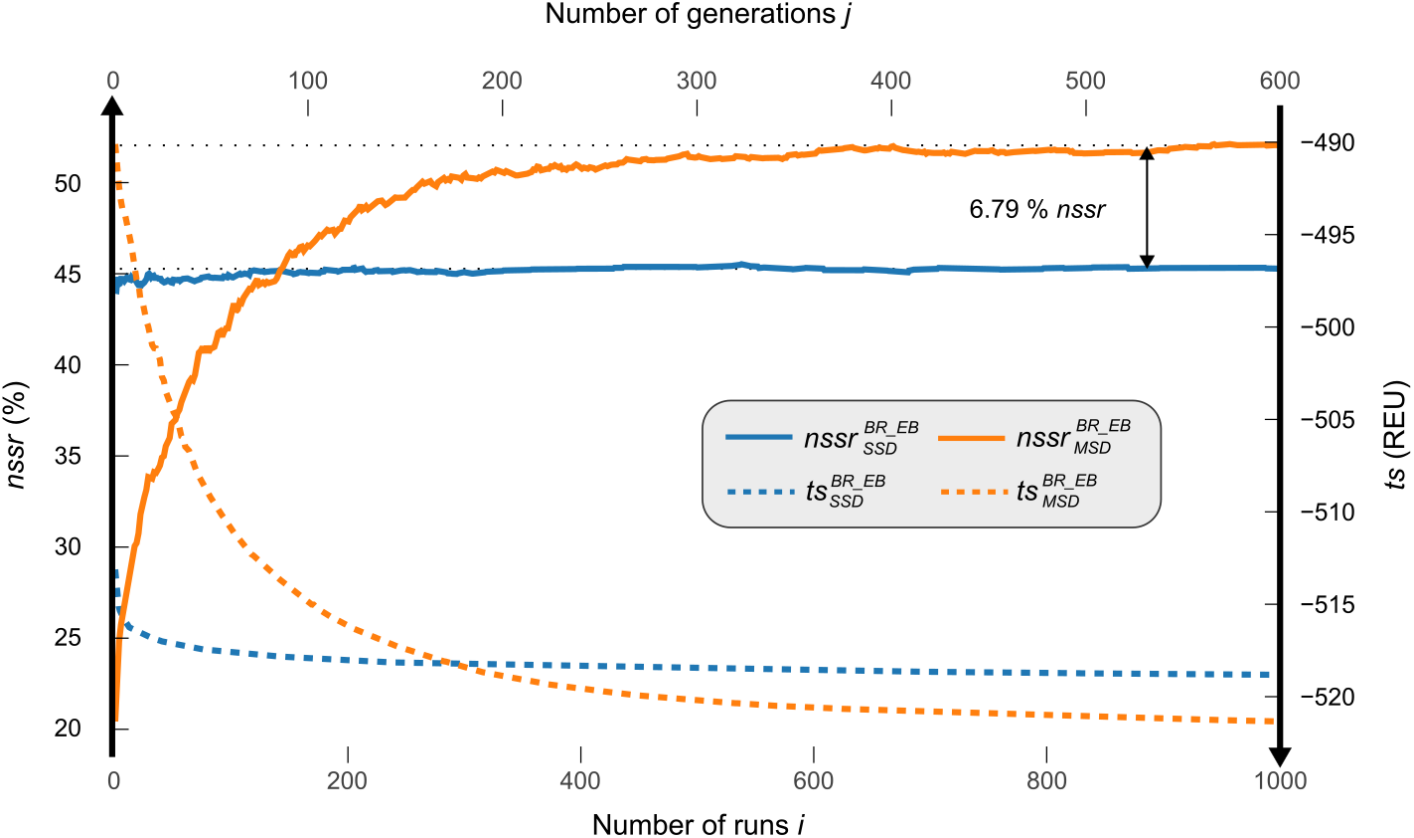
Convergence of SSD and MSD algorithms on the benchmark set *BR_EnzBench* enzdes (blue lines) was executed for 1000 runs *i* on all 20 conformations of each *prot*(*k*) from *BR_EnzBench*. For each number of runs *i*, the $ts{\;}_{SSD}^{BR\_EB}(i)$ value (dotted line) is the mean of the twenty lowest-energy sequences (Eq 9). The corresponding $nssr{\;}_{SSD}^{BR\_EB}(i)$ value (solid line) is the mean recovery value deduced from the same sequences (Eq 8). MSF:GA:enzdes (orange lines) was carried out for 600 generations *j* on all ensembles using a sequence population of 210. For each generation *j*, the $ts{\;}_{MSD}^{BR\_EB}(j)$ value (dotted line) is the mean of the five lowest-energy sequences of each of the four protein-specific ensembles (Eq 13). The corresponding $nssr{\;}_{MSD}^{BR\_EB}(j)$ value (solid line) is the mean recovery value deduced from the same sequences (Eq 12).

For SSD with enzdes, all conformations of each protein were considered independently and for each conformation, 1000 randomly seeded designs were performed. Design quality was assessed by means of the three parameters *nsr*, *nssr*, and *ts*. The respective values were averaged for each of the 16 *prot*(*k*) (Eqs 10 and 11) and are listed in Table 1. Additionally, the convergence of the design process was followed by monitoring the mean performance for each number *i* of design runs (Eqs 8 and 9); these values are plotted in Fig 3.

**Table 1:** Performance of SSD and MSD for individual proteins from *BR_EnzBench*.

PDB ID	*nsr* (%)		*nssr* (%)		*ts* (REU)	
	enzdes	MSF:GA: enzdes	enzdes	MSF:GA: enzdes	enzdes	MSF:GA: enzdes
1fzq	53.25	37.75	58.25	48.75	-325.16	-328.55
1hsl	34.74	33.95	60.00	59.47	-448.58	-447.39
1j6z	29.81	34.81	41.11	51.30	-771.96	-774.62
1n4h	28.80	28.80	53.00	59.40	-484.49	-488.89
1nq7	30.89	32.32	51.79	57.68	-506.56	-511.51
1opb	24.77	35.68	45.00	52.27	-307.57	-307.50
1pot	12.11	17.89	41.84	43.68	-613.10	-613.72
1urg	16.05	32.63	26.05	42.63	-796.85	-799.61
2b3b	24.41	41.47	32.35	50.59	-831.19	-831.17
2dri	21.58	25.79	42.89	55.26	-611.75	-613.74
2ifb	24.77	30.23	41.82	49.09	-305.08	-305.86
2q2y	38.70	39.13	48.48	56.52	-609.27	-611.17
2qo4	45.91	40.68	56.82	62.27	-271.47	-277.49
2rct	27.27	20.45	49.32	47.27	-317.51	-320.33
2rde	14.50	19.00	25.50	37.75	-463.52	-471.90
2uyi	38.26	37.61	47.17	56.09	-640.19	-641.01
**Average:**	**29.11**	**31.76**	**45.09**	**51.88**	**-519.02**	**-521.53**

*nsr*, *nssr*, and *ts* values were determined for each of the 16 proteins from *BR_EnzBench* after convergence of enzdes and MSF:GA:enzdes. For details, see Materials and Methods.

To conduct multi-state design by means of MSF:GA:enzdes, for each *prot*(*k*), the 20 conformations were divided into four ensembles $ens_{m}^{k}$ each containing five conformations. Note that the conformations that are combined in each of the ensembles $ens_{m}^{k}$ are unrelated, due to the stochastic approach of the Backrub algorithm. The GA was started on a population consisting of 210 sequences and stopped after 600 generations, because convergence was reached. Analogously, *nsr*, *nssr*, and *ts* values (Eqs 14 and 15) were determined for each MSD run and averaged for each of the 16 proteins. These results were added to Table 1. As above, the convergence of the GA was followed be monitoring the mean performance for each generation *j* (Eqs 12 and 13); these values are also plotted in Fig 3.

The protein-wise comparison (Table 1) indicates that in 10 out of the 16 cases, the *nsr* and in 13 out of all 16 cases, the *nssr* values of MSF:GA:enzdes designs are superior to the corresponding values of enzdes designs. MSF:GA:enzdes recovers on average a higher percentage of native residues (Δ *nsr* = 2.65%) and a higher percentage of similar residues (Δ *nssr* = 6.79%). Thus, with respect to the more adequate similarity measure *nssr*, MSD performs 15% better than SSD for this benchmark (*p* = 0.004, Wilcoxon signed rank test).

In addition, multi-state designs have slightly better energies (Δ *ts =* 2.51 REU), which is in contrast to the hIFABP results and is most likely due to the smaller ensemble size. Fig 3 reflects the differences in convergence speed of both algorithms and indicates that the better performance has its price: the MC optimization utilized by enzdes leads to acceptable design solutions even after a low number of runs. In contrast, the GA of MSF:GA:enzdes is slower and more than hundred generations are required to surpass the performance of the SSD algorithm. For this set of parameters, MSF:GA:enzdes required approximately five times the number of core hours needed by enzdes; further details of computational costs are given in S2 Text.

### The MSD concept is crucial for performance on *BR_EnzBench*

The sequence recovery reached for the hIFABP ensemble and for *BR_EnzBench* strongly suggests that MSF:GA:enzdes is superior to enzdes in more complex design applications. However, it was unclear to us, whether the different concepts (single-state versus multi-state) or the different optimizers (MC versus GA) contributed most to performance. Choosing an MSD approach increases computational cost, which has to be substantiated by making plausible that the choice of the optimizer is less important.

**Fig 4:**
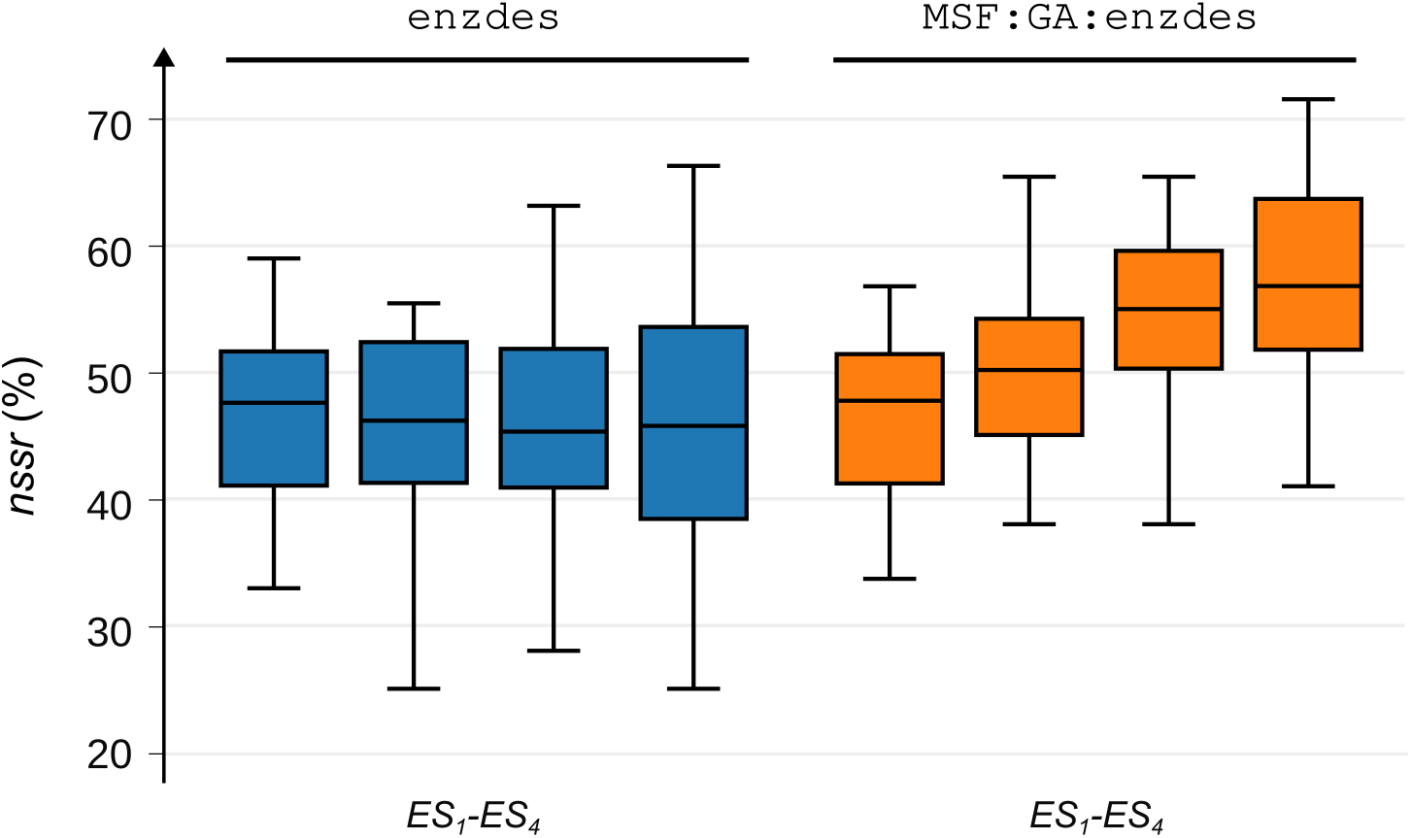
Performance of enzdes and MSF:GA:enzdes on a distinct grouping of conformations. Each of the sets *ES*_1_—*ES*_4_ contains a quarter of the conformations from *BR_EnzBench*, which were grouped according to their *nssr*_*MSD*_ values (Eq 16). *ES*_1_ contains all ensembles with the lowest and *ES*_4_ those with the largest recovery values. For each set *ES*_*i*_, the corresponding *nssr*_*SSD*_ (*ES*_*i*_) and *nssr*_*MSD*_ (*ES*_*i*_) values are represented by two boxplots. Left: performance of enzdes (blue boxplots), right: performance of MSF:GA:enzdes (orange boxplots). Whiskers indicate the lowest and the highest datum still within the 1.5 interquartile range.

The performance of MSF:GA:enzdes on *BR_EnzBench* was assessed ensemble-wise by determining the values $nssr_{MSD}(ens_{m}^{k})$, which were averaged (Eq 12). As these ensembles contain not more than five unrelated conformations each, the $nssr_{MSD}(ens_{m}^{k})$ values (Eq 16) vary due to the small sample size and one can sort for each *prot*(*k*) the four $ens_{m}^{k}$ on their $nssr_{MSD}(ens_{m}^{k})$ value. The result is a ranking $ens_{rank=u}^{k}$ (1 ≤ *u* ≤ 4) of the four ensembles and we created the set *ES*_1_ that contained the 16 ensembles (one for each *prot*(*k*)) with the lowest $nssr_{MSD}(ens_{m}^{k})$ value. Analogously, we compiled the sets *ES*_2_—*ES*_4_; consequently, *ES*_4_ consisted of those 16 ensembles that had the highest $nssr_{MSD}(ens_{m}^{k})$ value; for details see Materials and Methods. For these four sets *ES*_*i*_, we determined boxplots of the corresponding *nssr*_*SSD*_ and *nssr*_*MSD*_ values; see Fig 4.

The boxplots characterizing the SSD results are nearly identical; this finding indicates that the conformations allocated to the four sets *ES*_1_—*ES*_4_ give rise to a similar SSD performance. Moreover, the boxplots representing the *nssr*_*SSD*_ (*ES*_1_) and *nssr*_*MSD*_ (*ES*_1_) values are nearly identical (median values 47.60% and 47.76%), which indicates that the optimizer GA is not generally superior to MC. Additionally the continuous increase observed for the *nssr*_*MSD*_ (*ES*_1_) - *nssr*_*MSD*_ (*ES*_4_) - but not for the *nssr*_*SSD*_ (*ES*_1_) – *nssr*_*SSD*_ (*ES*_4_) values - supports the notion that it is the combination of conformations that strongly affects MSD performance. We thus conclude that the MSD approach - and not the optimizer - contributes most to the performance of MSF:GA:enzdes.

### *The residue preferences of* enzdes *and* MSF:GA:enzdes *differ*

Because Rosetta has a certain bias in recapitulating native residues [46], we assessed and compared the bias introduced by enzdes and MSF:GA:enzdes. For the assessment of the enzdes outcome, we selected the 13440 sequences representing the best designs on *BR_EnzBench* and determined *nssr*_*SSD*_ (*aa*_*j*_) values. This distribution represents for all amino acids *aa*_*j*_ the fraction of similar residues recovered at all design shell positions. Analogously, the distribution *nssr*_*MSD*_ (*aa*_*j*_) was computed that indicates the fraction of similar residues recovered by MSF:GA:enzdes; for details see Materials and Methods.

**Fig 5:**
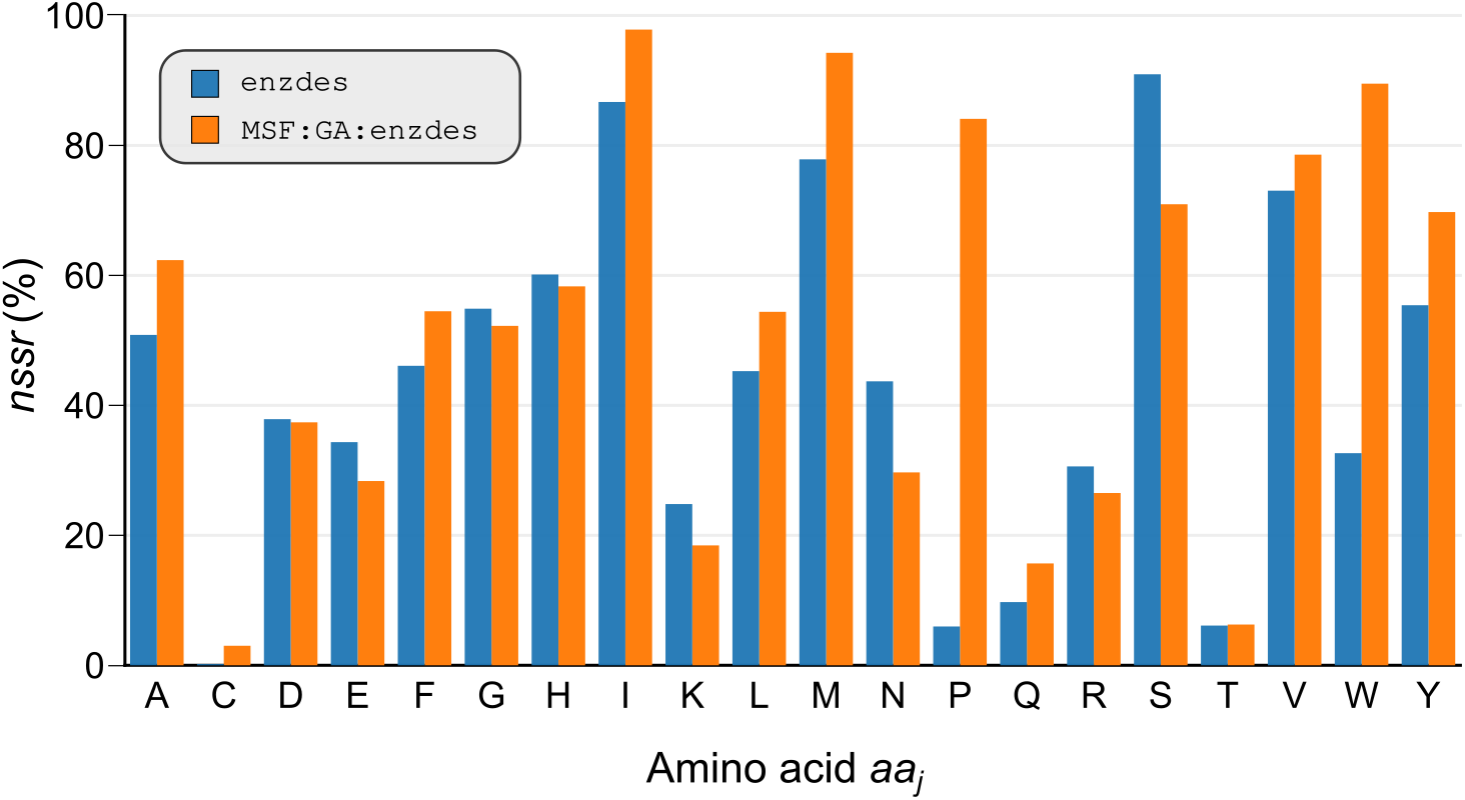
Recovery of design shell residues from *BR_EnzBench* by means of enzdes and MSF:GA:enzdes. The distributions *nssr*_*SSD*_ (*aa*_*j*_) (blue bars) and *nssr*_*MSD*_ (*aa*_*j*_) (orange bars) represent for each amino acid *aa*_*j*_ the *nssr* value (Eq 3) deduced from 13440 design sequences. These were created by enzdes or MSF:GA:enzdes for the benchmark proteins *BR_EnzBench*, respectively. *nssr* takes into account the recovery of all residues which are similar to the native *aa*_*j*_.

**Fig 6:**
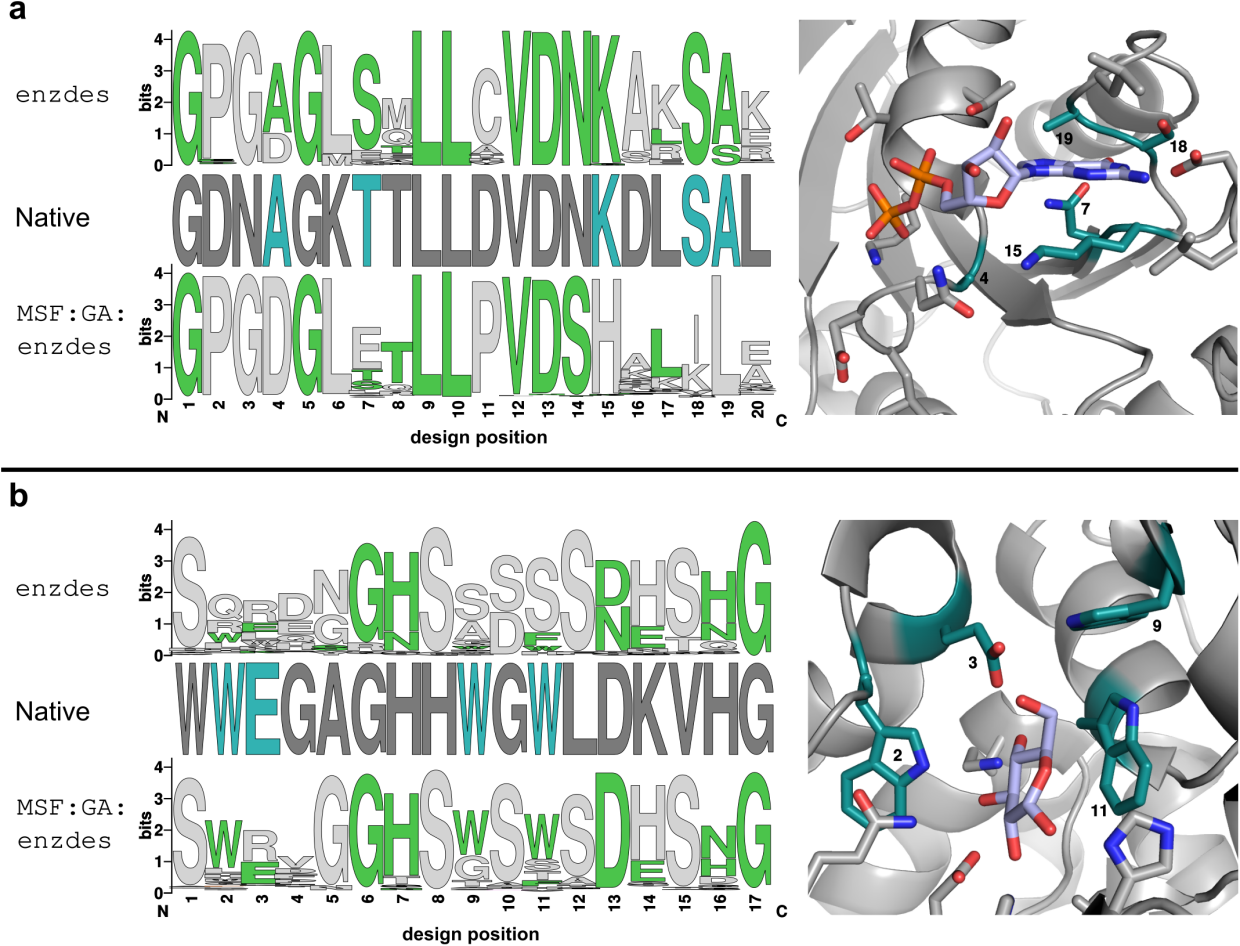
Recovery of two striking binding pockets by means of enzdes and MSF:GA:enzdes. **(a)** The 3D structure of the binding pocket of ARL3-GDP is shown on the right, the ligand GDP is colored light blue. The residues of the corresponding design positions are shown on the left (labeled “Native”). The sequence logos labeled enzdes and MSF:GA:enzdes represent for each design position the distribution of residues as generated by the corresponding protocols. Residues that are similar to the native ones are colored in green. In the native sequence, residues are colored in teal, if the outcome of the two protocols differs drastically. **(b)** The 3D structure of the binding pocket of the glucose binding protein is shown on the right; the bound glucose is colored light blue. Native residues and sequence logos are shown on the left and were prepared and colored as described for panel **(a)**.

The two distributions, which are plotted in Fig 5, indicate similar recovery rates that are below the optimal value of 100% for all residues. Generally, sequence recovery for large polar or charged residues (D, E, H, K, N, R, S) is low, which contributes to Rosetta’s weakness in accurately designing hydrogen bonds and electrostatics [47]. Interestingly, enzdes is slightly better in recovering polar and charged residues, whereas MSF:GA:enzdes clearly recovers a higher fraction of hydrophobic residues (A, F, I, L, P, V, W, Y). This general trend is most evident in the two benchmark proteins with the most extreme differences in their individual *nssr*_*SSD*_ and *nssr*_*MSD*_ values: ARL3-GDP (PDB ID 1fzq) is a distinct GTP binding protein [48] from *Mus musculus* and both the ligand and the native binding pocket are considerably polar. Fig 6A shows that enzdes correctly recovers the residues interacting with the guanine group (colored in teal) of GDP, while MSF:GA:enzdes is less successful. On the other hand, in the glucose binding protein (PDB ID 2b3b) from *Thermus thermophilus*, four tryptophan residues provide tight binding to glucose by shape complementarity. Fig 6B shows that MSF:GA:enzdes recovers three critical tryptophan residues (colored in teal) in most designs, whereas enzdes prefers small polar residues that do not provide tight packing.

We conclude that the representation of a protein by means of an ensemble improves hydrophobic packing but not the formation of polar interaction networks. Their design is considerably more difficult than hydrophobic packing due to the partially covalent nature of a hydrogen bond and the geometric requirements for orientations and distances [47, 49].

### Molecular dynamics simulations are well suited to create conformational ensembles

Molecular dynamics (MD) simulation is a well-established and reliable method for modeling conformational changes linked to the function of proteins [50]. Thus, MD provides an alternative to the Backrub approach for the generation of ensembles to be utilized in MSD. We were interested in assessing the designability of conformations resulting from unconstrained MD simulations of length 10 ns. In analogy to *BR_EnzBench*, we compiled the dataset *MD_EnzBench* consisting of 1000 conformations generated for each of the 16 benchmark apoproteins by means of YASARA [51]. Again, all design shell residues were replaced with alanine prior to design; see Materials and Methods.

To assess the structural variability of *MD_EnzBench* conformations, C_α_-RMSD values of design shell residues were determined in a protein-specific all-against-all comparison and then averaged. Analogously, the structural variability of *BR_EnzBench* conformations was determined. Interestingly, the variety of the binding pockets generated by the MD simulations is much larger than that generated by Backrub: the mean RMSD of *MD_EnzBench* is 0.62 Å and that of *BR_EnzBench* is 0.17 Å, which indicates that a 10 ns MD simulation generates an ensemble with higher structural diversity than the Backrub server.

**Fig 7:**
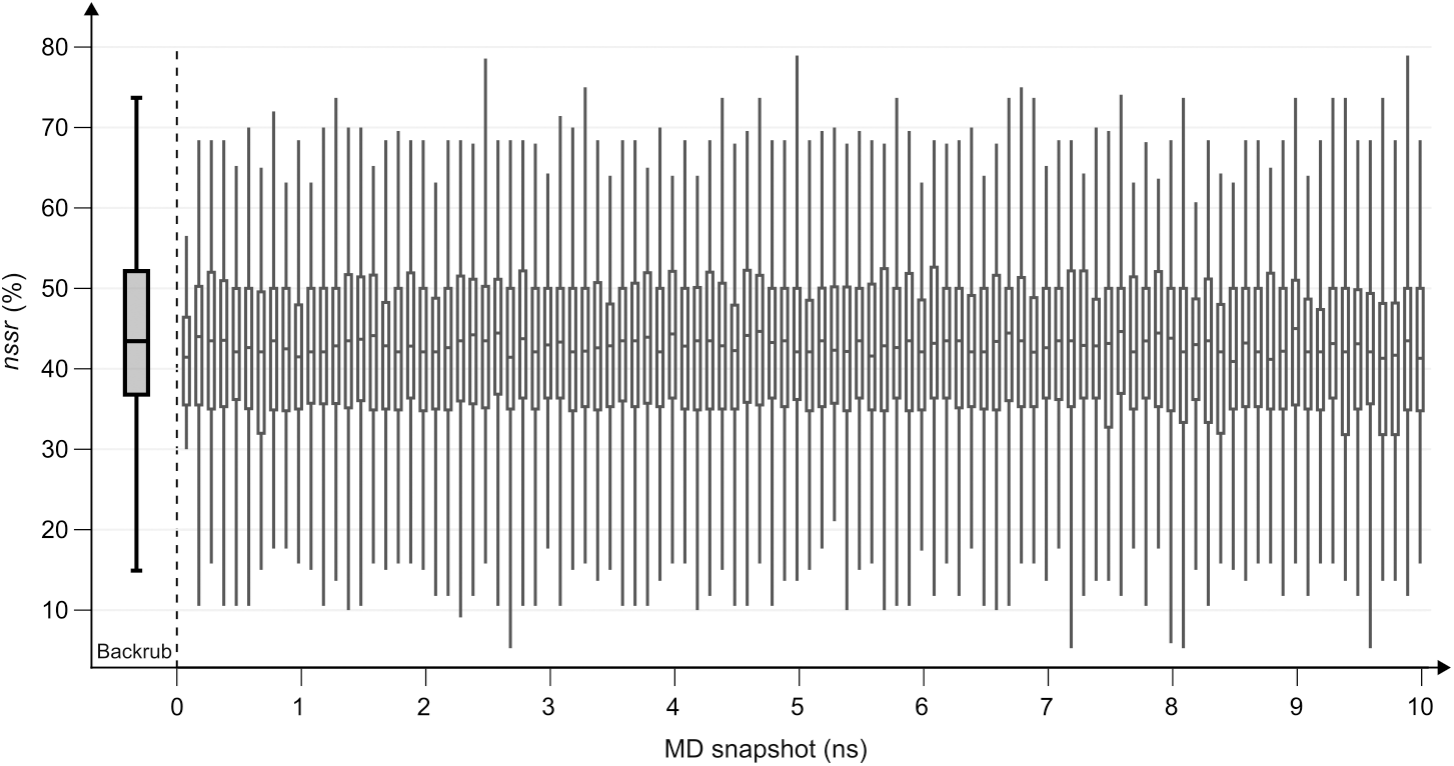
Single-state designability of *MD_EnzBench* conformations. Each of the 100 boxplots on the right represents 16 × 10 *nssr* values resulting from ten conformations generated by the MD simulation in a 100 ps interval for each of the 16 *prot*(*k*). As a control, the 16 × 20 $nssr{\;}_{SSD}^{BR\_EB}$ values of (single) enzdes designs generated for 20 protein-specific conformations from *BR_EnzBench* were summarized in a boxplot shown on the left (label Backrub). Whiskers indicate the lowest and the highest values of the 1.5 interquartile.

As a control of design performance, the 16 × 20 $nssr_{SSD}^{BR\_EB}(i=1)$ values of single enzdes designs generated for 20 protein-specific conformations from *BR_EnzBench* were summarized in a boxplot, which had a mean value of 43.88%. To assess the designability of the *MD_EnzBench* conformations, for each of the 1000 protein-specific conformations, one sequence was designed by means of enzdes and the resulting *nssr* values were averaged protein-wise. Fig 7 shows 100 boxplots each representing 16 × 10 *nssr* values resulting from ten conformations generated by the MD simulation in a 100 ps interval for each of the 16 *prot*(*k*). The mean of these *nssr* values is 42.53%, which testifies to a satisfying design performance, given that only one sequence was designed for each MD conformation. Moreover, the boxplots indicate that performance did not decrease for conformations generated at later phases of the MD simulation: the median *nssr*, and the first and third quartile of the most left and the most right boxplots are 42.10% [35.40%, 45.89%] and 42.24% [34.78%, 50.00%], respectively. In summary, these findings suggest that ensembles generated by MD feature higher conformational flexibility and appropriate *de novo* designability.

### A multi-state *de novo* design of retro-aldolases

The most convincing proof of concept for any CPD algorithm is the design of functionally active proteins. A non-natural reaction that is frequently chosen for enzyme design is the amine-catalyzed retro-aldole cleavage of 4-hydroxy-4-(6-methoxy-2-naphtyl)-2-butanone (methodol) into 6-methoxy-2-naphthaldehyde and acetone [52]. This multi-state reaction comprises the attack of an active site lysine side chain on the carbonyl group of the substrate to form a carbinolamine intermediate that is subsequently dehydrated to a protonated Schiff base. The latter is then converted to the reaction products by acid/base chemistry [53, 54]. The most active *de novo* retro-aldolase designs have been established on a jelly roll and several (βα)_8_-barrel proteins [55–57]. For comparison purposes, we selected the indole-3-glycerolphosphate synthase from *Sulfolobus solfataricus* (ssIGPS), a previously used thermostable (βα)_8_-barrel scaffold.

The native ligand was removed and the apoprotein was subjected to conformational sampling. Using the protocol validated with *MD_EnzBench*, three individual MD simulations were performed for 10 ns. A clustering of MD snapshots based on RMSD values helps to choose near-native conformations [58]. Thus, we used Durandal [59] to cluster the snapshots (conformations) generated with each MD run and picked four conformations from the largest cluster. These 3 × 4 conformations and the crystal structure of the apoprotein constituted the structural ensemble for the subsequent enzyme design.

Enzyme design generally starts with the assembly of a theozyme, which is a model for the proposed active site that is based upon the geometric constraints dictated by the expected transition state(s). To design retro-aldolase catalysis, we used a previously designed theozyme containing the carbinolamine reaction intermediate as transition state surrogate covalently bound to the catalytic lysine [56]. In addition, this theozyme contained an aspartate or a glutamate residue to function as general acid/base as well as a serine or a threonine residue to provide additional hydrogen-bonding interactions. Rosetta:match was applied to all conformations and created several thousand matched transition states (*mTS*) with catalytic triads K_*i*_-[D,E]_*j*_-[S,T]_*k*_ located at markedly different residue positions. A critical step of MSD is the compilation of the ensembles that are concurrently used as states. For enzyme design, ensembles *ens*_*mTS*_ of *mTS* are needed and we compiled them the following way: first, *mTS* judged as binding the transition state only weakly were discarded. Second, *mTS* derived from different conformations were added to the same *ens*_*mTS*_, if identical catalytic triads were located at matching residue positions. Thus, each *ens*_*mTS*_ contained a certain number of conformations accommodating the same catalytic triad. Third, the consistency of each *ens*_*mTS*_ was assessed by superposing the transition states and by comparing the corresponding conformations.

We chose 23 *ens*_*mTS*_ consisting of 4 to 13 conformations (states) and their design and repack shells were defined by merging the output created by enzdes:autodetect for all conformations. MSF:GA:enzdes was executed with each ensemble until energetic convergence; see S3 Text for details of the protocol. In brief, to assess the designs we compared active-site geometry as well as total and interaction energies and the best 100 variants were subjected to MD simulations of 10 ns length. For each variant, we analyzed in detail catalytic site geometries of 100 snapshots (see Materials and Methods) and nine variants named RA_MSD1 to RA_MSD9 were chosen for biochemical characterization; see S3 Text.

Because the catalytic efficiency and the conformational stability of initial designs are generally poor [60], further optimization is commonly performed by using either Foldit or other software tools to revert unnecessary mutations back to the native sequence of the scaffold [56], or by means of directed evolution [57]. However, we did not introduce subsequent stabilizing mutations into the sequences of RA_MSD1 to RA_MSD9 prior to a first experimental characterization. In doing so, we wanted to demonstrate the potential and also the limitations of multi-state designs.

**Fig 8:**
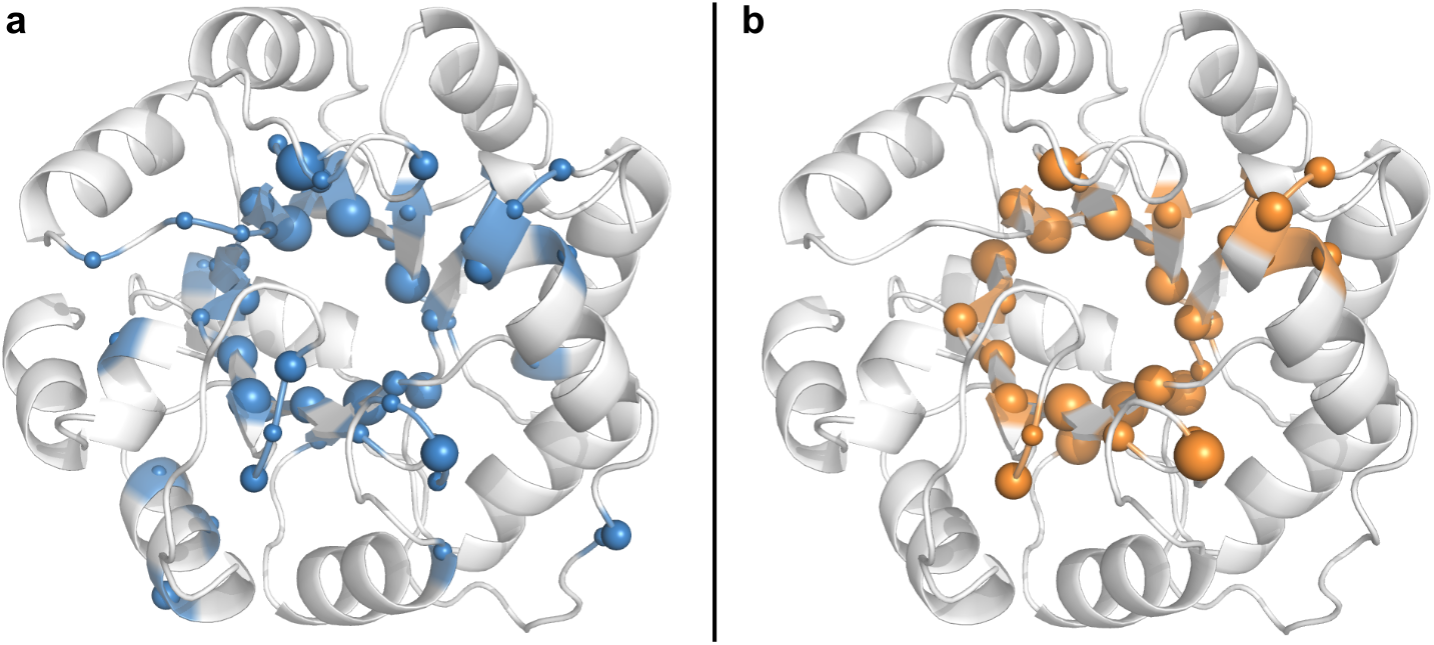
Mutations introduced into the IGPS scaffold to design retro-aldolase activity. (**a**) An overview of all mutations introduced in 42 previous designs subsumed in the set RA* which are listed in S3 Text. Blue spheres indicate residue positions and sphere diameters are proportional to the frequency of the mutations in comparison to the native IGPS sequence. (**b**) Ditto, for nine RA_MSD* designs, mutations are visualized by means of orange spheres.

For a comparison of these novel designs with previous ones, we compiled a list of 42 retro-aldolases RA* from the literature (see S3 Text) that were also created in the ssIGPS scaffold by means of Rosetta. These RA* sequences differ on average at 15 positions from the native ssIGPS sequence; in contrast, our nine RA_MSD* sequences contain on average 21 amino acid substitutions. Moreover, RA* sequences deviate on average from RA_MSD* sequences at 24 positions, and 18 substitutions distinguish the most similar pairs of variants (RA41 *versus* RA_MSD9 and RA90 *versus* RA_MSD8). Even a previous (RA114) and a new design (RA_MSD1), which share the same catalytic residues K210 and S110, differ at 25 positions. Thus, although we utilized the same TS and the same scaffold that was used for the design of RA114—RA120 [56], our MSD approach has generated a set of entirely novel catalytic sites located in the same shell as used for previous designs; see Fig 8.

### All initial MSD designs possess retro-aldolase activity but need further processing to improve solubility

The genes for RA_MSD1—RA_MSD9 were synthesized and expressed in *Escherichia coli* as fusion constructs with the gene for the maltose binding protein (MBP). The fusion proteins were purified with metal chelate affinity chromatography via their N-terminal hexa-histidine tags, resulting in high yields (50–150 mg protein/l expression culture). RA_MSD5 could be produced in soluble form also without MBP, whereas the other designs precipitated in the absence of the solubility enhancer. All designs showed modest catalytic activity with low substrate affinity, leading to conversion rates in the presence of 500 μM S-methodol ranging from 3 × 10^−7^ to 1.7 × 10^−5^ s^-1^ (Table 2). For the best designs, namely RA_MSD5 and RA_MSD7, the linear part of the substrate saturation curve was used to determine k_cat_/K_M_ values of 3.47 × 10^−2^ and 1.41 × 10^−2^ M^-1^s^-1^ (S1 Fig; Table 2), which are similar to the values obtained for RA114 - RA120 [46]. Moreover, the RA_MSD5 designs with and without MBP displayed virtually the same k_cat_/K_M_ values, excluding an influence of the solubility enhancer on activity.

**Table 2:** MSD proteins and their retro-aldolase activity.

Name	Catalytic triad	Number of mutations compared to ssIGPS	Conversion rate (s^-1^)	k_cat_/K_M_ (M^-1^s^-1^)
RA_MSD1	K210 D131 S110	21	8.08 × 10^−7^	ND
RA_MSD2	K210 D131 S110	22	3.14 × 10^−7^	ND
RA_MSD2.4	K210 D131 S110	26	1.23 × 10^−6^	ND
RA_MSD2.5	K210 D131 S110	29	1.49 × 10^−6^	ND
RA_MSD3	K210 D131 S110	22	2.60 × 10^−6^	ND
RA_MSD4	K51 E53 S83	20	3.03 × 10^−6^	ND
RA_MSD5	K51 E53 S83	21	1.69 × 10^−5^	3.47 × 10^−2^
RA_MSD6	K231 E53 S83	25	2.82 × 10^−6^	ND
RA_MSD7	K231 E131 T159	18	8.33 × 10^−6^	1.41 × 10^−2^
RA_MSD8	K231 E131 T159	18	5.61 × 10^−6^	ND
RA_MSD9	K231 E53 T83	19	7.55 × 10^−7^	ND

The catalytic triad designed for nine proteins (RA_MSD1—RA_MSD9) is specified in the second column. The third column gives the number of residue exchanges compared to the native sequence of ssIGPS. The fourth column lists the conversion rates (rate of product formation divided by the enzyme concentration) in the presence of 500 μM S-methodol. The last column gives the catalytic efficiency k_cat_/K_M_ as determined for RA_MSD5 and RS_MSD7 from the linear part of substrate saturation curves; see S1 Fig. ND: not determined.

Due to the intentionally omitted step of secondary protein stabilization following the initial design process, eight of our nine designs were insoluble without MBP. We wanted to test whether protein stabilization would result in higher activity. Accordingly, we attempted to improve the stability of RA_MSD2, which has the lowest activity of all designs (Table 2), by using the fully automated *in silico* method offered by the PROSS webserver [61]. The six conformations of RA_MSD2 were individually submitted to PROSS and the corresponding output sets that contained 6 to 21 stabilizing mutations were merged to five consensus sequences; see S3 Text, Table B1. Variants RA_MSD2.4 and RA_MSD2.5 that contained the highest number of stabilizing mutations, could be produced in soluble form without MBP and were purified with high yield (about 25 mg protein/l expression culture). Activity measurements showed, however, that the additional stabilizing exchanges did not drastically improve the conversion rate of RA_MSD2; see Table 2.

In summary, our results show that MSD (based on a structural ensemble) is comparably successful as SSD (based on a single structure) for establishing retro-aldolase activity on a thermostable (βα)_8_-barrel scaffold, indicating that this particular reaction requires only a limited degree of conformational flexibility. However, catalysis is often linked to conformational transitions which can only be captured by MSD approaches. Moreover, in contrast to SSD, MSD offers a broader functionality and is for example also suited for more challenging tasks like negative design.

## Materials and methods

### Benchmark datasets *BR_EnzBench* and *MD_EnzBench*

Two subsets of the scientific sequence recovery benchmark of Rosetta [42] were generated that contain 20 specifically prepared conformations of 16 proteins *prot*(*k*) with bound ligand. In order to exclude an erroneous conformational sampling, missing residues were reconstructed by using YASARA:loop_modeling [62] and the respective native sequences. Additionally, all ligands were removed prior to the conformational sampling of the resulting apoproteins.

The dataset *BR_EnzBench* was created by using the BackrubEnsemble method of the Backrub server [43] to compute a conformational ensemble of 20 structures for each apoprotein. The second benchmark dataset *MD_EnzBench* was deduced from MD simulations of length 10 ns generated with YASARA (version 14.7.17) and the YAMBER3 force field, which has been parameterized to produce crystal structure-like protein coordinates [51]. For each of the 16 apoproteins, 1000 conformations were sampled at an interval of 10 ps. After sampling, the native ligands were re-introduced in all conformations of both subsets by means of PyMOL:superpose [63] and the respective apoproteins.

For the corresponding holoproteins of *BR_EnzBench* and *MD_EnzBench*, the same design and repack shells were utilized. These were determined protein-wise for each of the *BR_EnzBench* conformations by means of Rosetta:enzdes:autodetect and merged. In all conformations, design shell residues were replaced with alanine and prior to design, all conformations were energy-minimized by means of Rosetta:fastrelax with backbone constraints. Parameters of MD simulations, Rosetta:fastrelax, and the composition of design and repack shells are listed in S2 Text.

### Genetic algorithm and fitness function

The first generation of the 210 sequences consisted of the given seed sequence and 209 mutants each with a randomly introduced single point mutation. During each generation cycle, half of the population was replaced with sequences *seq*_*i*_ generated by means of single point mutations and recombination. The replaced sequences were those with worst fitness values *fitness*(*seq*_*i*_), which were computed for MSF:GA:enzdes according to:

$$fitness(seq_{i})=\frac{1}{n}\sum_{l=1}^{n}ts_{l}(seq_{i})$$ (2)

Here, *n* is the number of states (*e*. *g*. conformations *s*_1_, …, *s*_*n*_ of a given *prot*(*k*)) and *ts*_*l*_ is the Rosetta total score for a sequence given a state *l*. In all equations, *ts* values are given in REU.

### Computing the native sequence similarity recovery

For a given pair of residues *aa*_1_, *aa*_2_ the *nssr* value was deduced from the scores of the BLOSUM62-matrix [64] as follows:

$$nssr(aa_{1},aa_{2})=\;\{\begin{array}{ll} \;1 & \mathrm{if}\;\mathrm{BLOSUM62}(aa_{1},aa_{2})>0\\ \;0 & \mathrm{else} \end{array}$$ (3)

For a given pair of sequences *seq*_1_, *seq*_2_ of length *n*, the *nssr* value was determined as the mean value deduced for residue pairs *seq*_1_[*i*], *seq*_2_[*i*]:

$$nssr(seq_{1},seq_{2})=\frac{1}{n}\sum_{i=1}^{n}nssr(seq_{1}[i],seq_{2}[i])$$ (4)

For a given set of design solutions *ds* = {*seq*_1_,…,*seq*_*m*_} and a native sequence *seq*_*nat*_, the value *nssr*(*ds*,*seq*_*nat*_) was computed according to:

$$nssr(ds,seq_{nat})=\frac{1}{m}\sum_{i=1}^{m}nssr(seq_{i},seq_{nat})$$ (5)

### Assessing design performance on hIFABP

The data set with PDB ID 2mji contains ten conformers of hIFABP and the bound ligand ketorolac; this ensemble has been deduced by means of solution NMR [65]. The set was downloaded from PDB and the ligand was parameterized using Rosetta:molfile-to-params [66]. Next, each of the ten conformations was energy-minimized via Rosetta:fastrelax with constraints. To obtain consistent design and repack shells, the shells determined by Rosetta:enzdes:autodetect for each conformation were merged.

For SSD, enzdes was applied to each of the ten initial conformations *conf*(*l*) (1 ≤ *l* ≤ 10). Using the default MC optimization and the parameter set *ps*_*enzdes*, sequences *seq*_*l*_ (*i*) were generated by means of 1000 randomly seeded *runs*_*l*_ (*i*) (1 ≤ *i* ≤ 1000). In order to control the convergence of the design process and for performance comparison, the $seq_{l}^{*}(i)$ with the best total score (*ts*) were chosen from *seq*_*l*_ (1,…,*i*) for each *l* and each *i*. Finally, the mean of the ten *ts* values was determined as a measure of design quality $ts{\;}_{SSD}^{hIFABP}(i)$ reached in *i* SSD runs:

$$ts_{SSD}^{hIFABP}(i)\;=\;\frac{1}{10}\sum_{l=1}^{10}ts(seq_{l}^{*}(i))$$ (6)

For MSD, all ten conformations *conf*(*l*) were considered as states and MSF:GA:enzdes was executed for 800 generations (*i*. *e*. design cycles) on a population consisting of 210 sequences with parameters *ps_msf_enzdes*. The initial population was seeded with the native sequence. The sequences representing a generation *j* were ranked with respect to *ts* values and the ten top scoring sequences $seq_{l}^{t}(j)$ (1 ≤ *t* ≤ 10) were stored in order to allow for the subsequent performance comparison. Finally, the mean of the 10 × 10 *ts* values was determined as a measure of design quality $ts{\;}_{MSD}^{hIFABP}(j)\;$ reached in *j* MSD generations:

$$ts_{MSD}^{hIFABP}(j)\;=\;\frac{1}{100}\sum_{l=1}^{10}\sum_{t=1}^{10}ts(seq_{l}^{t}(j))$$ (7)

Further details of the analysis can be found in S2 Text; it lists parameters of Rosetta:fastrelax and the design protocol, and the composition of the design and repack shell.

### Assessing design performance on *BR_EnzBench*

For SSD, enzdes was applied to each of the 20 initial conformations *conf*(*l*) (1 ≤ *l* ≤ 20) of each *prot*(*k*) (1 ≤ *k* ≤ 16) from *BR_EnzBench*. Using default MC optimization and the parameter set *ps_enzdes* (see S2 Text), sequences *seq*_*k*,*l*_ (*i*) were generated by means of 1000 randomly seeded *runs*_*k*,*l*_ (*i*) (1 ≤ *i* ≤ 1000). In order to control the convergence of the design process and for performance comparison, those $seq_{k,l}^{*}(i)$ having the best *ts* value were chosen from *seq*_*k*,*l*_ (1,…,*i*) for each *k*, *l*, and *i*. Finally, mean performance reached in *i* SSD runs was measured by means of the *score* ∈ {*nsr*,*nssr*}, where *nsr* is the native sequence recovery and *nssr* is the native sequence similarity recovery:

$$score{\;}_{SSD}^{BR\_EB}(i)\;=\;\frac{1}{320}\sum_{k=1}^{16}\sum_{l=1}^{20}score(seq_{k,l}^{*}(i),seq_{nat}^{k})$$ (8)

$$ts_{SSD}^{BR\_EB}(i)\;=\;\frac{1}{320}\sum_{k=1}^{16}\sum_{l=1}^{20}ts(seq_{k,l}^{*}(i))$$ (9)

Here, $seq_{nat}^{k}$ is the native sequence of *prot*(*k*), and *ts* is the total score. To score SSD performance reached for one *prot*(*k*), the final score values were averaged over all conformations:

$$score{\;}_{SSD}^{BR\_EB}(k)\;=\;\frac{1}{20}\sum_{l=1}^{20}score(seq_{k,l}^{*}(1000),seq_{nat}^{k})$$ (10)

$$ts{\;}_{SSD}^{BR\_EB}(k)\;=\;\frac{1}{20}\sum_{l=1}^{20}ts(seq_{k,l}^{*}(1000))$$ (11)

To assess the performance of MSD, each of the 20 conformations of a *prot*(*k*) was assigned to an ensemble $ens_{m}^{k}$ (1 ≤ *m* ≤ 4) consisting of five conformations each. These five conformations were considered as states and MSF:GA:enzdes was executed for 600 generations on a population consisting of 210 sequences with parameter set *ps_msf_enzdes* (see S2 Text). The initial population was seeded with an all-alanine sequence. The sequences representing a generation *j* were ranked with respect to *ts* values and the five top scoring sequences $seq_{k,m}^{t}(j)$ [1 ≤ *t* ≤ 5] were stored in order to allow for the subsequent performance comparison. Finally, mean performance values reached in *j* MSD generations were determined according to:

$$score{\;}_{MSD}^{BR\_EB}(j)\;=\;\frac{1}{320}\sum_{k=1}^{16}\sum_{m=1}^{4}\sum_{t=1}^{5}score(seq_{k,m}^{t}(j),seq_{nat}^{k})$$ (12)

$$ts{\;}_{MSD}^{BR\_EB}(j)\;=\;\frac{1}{320}\sum_{k=1}^{16}\sum_{m=1}^{4}\sum_{t=1}^{5}fitness(seq_{k,m}^{t}(j))$$ (13)

Here, $seq_{nat}^{k}$ is the native sequence of *prot*(*k*), *score* ∈ {*nsr*,*nssr*} is a sequence recovery, and $fitness(seq_{k,m}^{t}(j))$ is the mean *ts* score (Eq 2, *n* = 5) of a given sequence over the five conformations belonging to ensemble $ens_{m}^{k}$. To score MSD performance reached for one *prot*(*k*) after 600 generations, the final score values were averaged over all ensembles:

$$score{\;}_{MSD}^{BR\_EB}(k)\;=\;\frac{1}{20}\sum_{m=1}^{4}\sum_{t=1}^{5}score(seq_{k,m}^{t}(600))$$ (14)

$$ts{\;}_{MSD}^{BR\_EB}(k)\;=\;\frac{1}{20}\sum_{m=1}^{4}\sum_{t=1}^{5}fitness(seq_{k,m}^{t}(600))$$ (15)

### Grouping ensembles by MSD performance

The 20 conformations of a given protein *prot*(*k*) from *BR_EnzBench* belong to one of four ensembles $ens_{1}^{k}$ - $ens_{4}^{k}$. The performance values $nssr_{MSD}(ens_{m}^{k})$ were determined for each *prot*(*k*) and each $ens_{m}^{k}$ according to:

$$nssr_{MSD}^{}(ens_{m}^{k})\;=\;\frac{1}{5}\sum_{t=1}^{5}nssr(seq_{k,m}^{t}(600),seq_{nat}^{k})$$ (16)

Here, $seq_{nat}^{k}$ is the native sequence of *prot*(*k*). The values $nssr_{MSD}(ens_{m}^{k})$ were used for a ranking $ens_{rank=u}^{k}$ (1 ≤ *u* ≤ 4) of the four ensembles such that $ens_{rank=1}^{k}$ is the one with the lowest $nssr_{MSD}(ens_{m}^{k})$ value and $ens_{rank=4}^{k}$ that with the largest one. Having ranked the ensembles of all *prot*(*k*), sets of ensembles were created such that the set $ES_{1}=\overset{16}{\underset{k=1}{\cup }}ens_{rank=1}^{k}$ contained those ensembles that performed worst and $ES_{4}=\overset{16}{\underset{k=1}{\cup }}ens_{rank=4}^{k}$ those that performed best and the intermediates with *rank* = 2 and *rank* = 3 performed accordingly. For these four sets *ES*_*i*_, boxplots of the corresponding *nssr*_*SSD*_ and *nssr*_*MSD*_ values were determined.

### Choosing sequences for the analysis of the sequence differences

In order to assess the amino acid composition of the enzdes outcome, the 42 *seq*_*k*,*l*_ (1,…,1000) with optimal *ts* values were identified for each of the 20 conformations *l* of all *prot*(*k*) ∈ *BR*_*EnzBench*. For these 16 × 840 sequences $seq_{SSD}^{k}$, the values $nssr(seq_{SSD}^{k}[i],seq_{nat}^{k}[i])$ were determined (Eq 3) by comparing design shell and native (*nat*) residues *i*. The distribution *nssr*_*SSD*_ (*aa*_*j*_) represents for all amino acids *aa*_*j*_ their recovered similarity at all design shell positions.

To assess the amino acid composition for the MSF:GA:enzdes outcome, the 16 × 4 × 210 sequences $seq_{MSD}^{k}$ of the final populations (*i*. *e*. all *seq*_*k*,*m*_ (600)) generated for the four ensemble groups of each *prot*(*k*) ∈ *BR*_*EnzBench* were used to determine the values $nssr(seq_{MSD}^{k}[i],seq_{nat}^{k}[i])$. The distribution *nssr*_*MSD*_ (*aa*_*j*_) represents for all amino acids *aa*_*j*_ their recovered similarity at all design shell positions.

### Multi-state design of retro-aldolases

The scaffold protein indole-3-glycerol phosphate synthase from *S*. *solfataricus* (ssIGPS, PDB ID 1a53), was downloaded from PDB and the ligand IGP was removed. To generate a structural ensemble, three MD simulations were performed with the apoprotein for 10 ns by means of YASARA and the YAMBER3 force field. Using Durandal:smart-mode:semi-auto[0.03. 0.20], the snapshots of each trajectory were clustered individually and four conformations were chosen from the largest cluster. These 12 conformations and the crystal structure of 1a53 were used for matching the transition state (TS) and grafting the theozyme of the retroaldol reaction [56] by means of Rosetta:match. Each of the resulting matched transition states (*mTS*) consisted of a catalytic triad K_*i*_-[D,E]_*j*_-[S,T]_*k*_ at three residue positions *i*, *j*, *k* that occured in one of the 13 conformations.

Ensembles *ens*_*mTS*_ of *mTS* used as input for MSF:GA:enzdes were generated as follows: first, *mTS* were discarded that were classified as weak TS binders or TS destabilizers. For example, matches with catalytic residues near the protein surface were eliminated. Second, *mTS* were grouped according to the composition and localization of the catalytic triad and those ensembles were selected that were compatible with most of the 13 conformations. Third, *ens*_*mTS*_ were assessed with respect to the structural similarity of the superposed theozymes. In total, 23 ensembles *ens*_*mTS*_ containing 4 up to 13 conformations were chosen. For each *ens*_*mTS*_, the design and repack shells were defined by merging the outcome of Rosetta:enzdes:autodetect for all corresponding conformations and MSF:GA:enzdes was executed on a population of 210 sequences that were seeded with the native sequence of ssIGPS. At convergence, the design process was stopped, which was the case after 97 to 710 generations. S3 Text lists more details of the design procedure like parameters of MD simulations and of Rosetta:match, and the specification of the TS.

### Evaluation of multi-state design solutions

After MSD of retro-aldolases, the designs were filtered by *ts* values and active-site geometry. The best 100 designs were selected for 10 ns MD simulations in water and for one conformation of each design ensemble, 100 snapshots were generated. Two simulations were performed; the first one was based on the enzyme/TS complex. As a control, the second MD simulation was based on the enzyme/substrate complex and the substrate methodol was created by deleting the lysine-substrate bond of the TS. For each trajectory, catalytic distances, angles and torsion angles were plotted as boxplots and used to assess the designs; see S3 Text.

### PROSS stabilization

Variant RA_MSD2 was chosen for solubilization experiments and all six conformations *conf*(*l*) of the corresponding ensemble were submitted to the PROSS server [61], which was used with default settings allowing for mutations at all positions. For each input *conf*(*l*), PROSS provided seven mutated sequences *mut_seq*_*l*_(*i*) (1 ≤ *i* ≤ 7) containing an increasing number of putatively stabilizing mutations. For each *i* (degree of stabilization), an MSA that contained all sequences *mut_seq*_*l*_(*i*) computed for all *conf*(*l*) was generated and weblogo [67] was used to determine a sequence logo. Finally, consensus residues deduced from the sequence logos were accepted as mutations at sites that did not interfere with the catalytic center. All sequence logos are shown in S3 Text.

### Cloning, gene expression, and protein purification

The genes encoding the designed retro-aldolases were optimized for *E*. *coli* codon usage and ordered as synthetic gene strings from Life Technologies. Cloning was performed via BsaI restriction sites into pET28a (Stratagene) and pMalC5T (New England Biolabs) plasmids specifically modified for this method of cloning. Both vectors fuse an N-terminal his_6_-tag to the target proteins, pMalC5T additionally adds MBP. The cloning method is derived from golden gate cloning [68]. Details of plasmid construction and cloning procedure will be published elsewhere. *E*. *coli* BL21 Gold cells were transformed with the resulting plasmids. The cells were grown in Luria broth with 50 μg/ml kanamycin or 150 μg/ml ampicillin for pET28 constructs and pMAL constructs, respectively. At a cell density of OD_600_ = 0.5 protein production was induced by addition of 0.5 mM isopropyl-β-thiogalactopyranoside. After growth over night at 20°C the cells were harvested by centrifugation (Avanti J-26 XP, JLA 8.1000, 15 min, 4,000 rpm, 4°C). Cell pellets were resuspended in 50 mM Tris/HCl buffer (pH 7.5) with 300 mM NaCl. Cells were lysed by sonication (Branson Sonifier W-250D, amplitude 65%, 3 min, 2 s pulse/2 s pause). Cell debris was removed by centrifugation (Avanti J-26 XP, JA 25.50, 30 min, 14,000 rpm, 4°C) and soluble proteins were purified by nickel chelate affinity chromatography (GE Healthcare, HisTrap FF crude). The proteins were eluted with 50 mM Tris/HCl (pH 7.5) containing 300 mM NaCl using a gradient of 10–500 mM imidazole. Fractions containing sufficiently pure protein were pooled and excess imidazole was removed by dialysis against 50 mM Tris/HCl (pH 7.5) buffer containing 100 mM NaCl. Protein concentrations were determined by absorbance spectroscopy (NanoDrop One, Thermo Fisher) using extinction coefficients determined by the Expasy:ProtParam webtool.

### Activity assay

Retro-aldolase activity of the designs (30–50 μM) was measured at 25°C in 50 mM Tris/HCl (pH 7.5), 100 mM NaCl and 5% (v/v) dimethyl sulfoxide (for substrate solubility) by following the formation of the fluorescent product 6-methoxy-2-naphthaldehyde from non-fluorescent S-methodol (70% ee). The substrate was synthesized as described in S3 Text. Fluorescence was measured in a Mithras LB 940 plate reader (λ_ex_ = 355 nm, λ_em_ = 460 nm) using black 96 well micro plates. The concentrations of product were determined with the help of a calibration curve. For the determination of conversion rates, each measurement was repeated four times, for k_cat_/K_M_ determinations all points were measured as triplicates. The wild-type scaffold protein ssIGPS and the solubility tag MBP served as negative controls and did not show any detectable activity. Further control measurements showed that conversion rates in the presence of 5% (v/v) dimethyl sulfoxide were identical to those in 3% acetonitrile, which has been used for the characterization of other retro-aldolase designs [46].

## Supporting information

S1 Text Technical details, availability, and how to run MSF. (PDF)

S2 Text Details of software validation, benchmark datasets and their compilation. (PDF)

S3 Text Multi-state approach to design retro-aldolases. (PDF)

S1 Fig Steady-state enzyme kinetics of RA_MSD5 and RA_MSD7. Due to the low affinity of the two designs for S-methodol, only the linear part of the substrate saturation curves could be determined. The slopes yielded catalytic efficiencies (k_cat_/K_M_) of 3.47 × 10^−2^ and 1.41 × 10^−2^ M^-1^s^-1^ for RA_MSD5 and RA_MSD7, respectively.
(PDF)
