# Supplementary material for: Rosetta:MSF: a modular framework for multi-state computational protein design
Source: PLoS Comput Biol. 2017 Jun 12;13(6):e1005600. doi: 10.1371/journal.pcbi.1005600 (PMC5484525; doi:10.1371/journal.pcbi.1005600)
Supplement: S1 Text — (PDF) [file pcbi.1005600.s001.pdf]

Rosetta:MSF: a modular framework for multi-state computational protein design

Patrick Löffler, Samuel Schmitz, Enrico Hupfeld, Reinhard Sterner, Rainer Merkl

Technical details, availability and how to run MSF

This document provides more details of the MSF implementation as well as information about the availability, compilation, and typical run time options.

I. Technical details

MSF has been integrated as an additional protocol into Rosetta and is purely written in C++11. It was our aim to implement a framework that significantly reduces the development efforts of augmenting arbitrary protocols with multi-state design (MSD) capability. Thus, MSF bundles a number of generalized (template-)classes responsible for distributing tasks to available computational resources, establishing MPI-based communication and task synchronization [1], as well as initialization and execution of Rosetta protocols. To generate candidate sequences, MSF utilizes Rosetta's own genetic algorithm (GA). For the initial implementation, two single-state protocols from Rosetta were integrated into the framework: enzdes provides ligand binding protein/enzyme design functionality by repacking and redesigning residues around the binding/active site and allowing for the optimization of catalytic contacts; AnchoredDesign can create new protein-protein interactions by using information from known interactions of the target partner protein. The resulting applications msf_ga_enzdes and msf_ga_anchored are ready to use and expose all existing options of enzdes and AnchoredDesign in addition to genetic algorithm protocol options to the user. Those applications exemplify the integration of typical SSD protocols into MSF. Compared to MPI_MSD or RECON, this integration requires less development efforts because of the modularity of MSF.

MSF was written from scratch and designed with high maintainability and flexibility in mind. It allows the easy access to core elements due to a global allocation system managing the instantiation of polymorphic key classes. This means, that most important functions of the multi-state design pipeline are exposed to the application and may be modified for each application individually without breaking compatibility to existing applications. To accommodate MSD, a combined score is calculated according to a so-called "dynamic aggregation function" (DAF, introduced by MPI_MSD). It consists of mathematical operations used to weight individual states and this is why it does also support positive and negative design. Based on this DAF, the fitness of all sequences is evaluated for all given states. MSF was designed with optimal scalability: for assessing the fitness of the candidate sequences within the "inner routine", the upper limit of processes to be utilized by MPI_MSD is the number of *states* under study. In contrast, MSF can make use of (*states* × *population size*) processes, which is a tremendous speed-up, because populations commonly consist of more than 100 sequences seq_i.

II. Instructions to install, compile and run MSF

MSF has been developed and tested for Rosetta's weekly release 2015.19.57819. The branch named SamuelSchmitz/msf_2015.19.57819 contains two applications, namely `msf_ga_enzdes` and `msf_ga_anchored`. It can be accessed by RosettaCommons developers at:
https://github.com/RosettaCommons/main/tree/SamuelSchmitz/msf_2015.19.57819

The integration of `MSF:GA:enzdes` into Rosetta's master branch is current work and aims at providing functionality for upcoming releases.

MSF requires the compilation of Rosetta with MPI support; for example by executing

```
./scons.py mode=release extras=mpi msf_ga_enzdes msf_ga_anchored
```

Command line options of the genetic algorithm

The following command line options define the parameters of the genetic algorithm:

**Option (MSF namespace)**	**Description**	**Default**
`checkpoint_write_interval`	Write checkpoint every x generations	`1`
`checkpoint_prefix`	Path to checkpoint files	
`darwin_resume`	Resume from checkpoint files	`false`
`fill_gen1_from_seed_sequences`	Initialize GA from distinct sequences	`false`
`fraction_by_recombination`	Fraction of the population that is recombined every generation	`0.05`
`generations`	Number of generations to be evolved	`0`
`pop_size`	Population size	`0`
`resfile_tmpdir`	Path to temporary resfiles	
`seed_sequences`	List of sequences separated by ","	
`seed_sequences_from_input_pdb`	Generate initial population based on sequence from the input structure	
`seed_sequence_using_correspondence_file`	Extract sequence from design shell	`false`

Options required for multi-state design

Current MSF applications require the setup of an *entity_resfile* and *fitness_file*, which replicate the functionality of MPI_MSD's counterparts. See the documentation of MPI_MSD for these functions:
https://www.rosettacommons.org/docs/latest/application_documentation/design/mpi-msd

**Option (MSF namespace)**	**Description**	**Default**
`entity_resfile`	Defines the size of the design shell	
`fitness_file`	Path to the DAF file, which defines how to calculate an ensemble score	
`read_states_on_demand`	Load state structures on demand from disk; saves memory when a large number of states are used	`false`

Example command to run the application **msf_ga_enzdes**:

A typical call of `msf_ga_enzdes` is the following one:

```
mpirun –np num_cpu \
$ROSETTA_ROOT/main/source/bin/msf_ga_enzdes.mpi.linuxgccrelease \
@flags
```

A reasonably chosen value for the number of processes is the number of states multiplied with half the size of the GA population. Please refer to the options table above to fill the `flags` file. Example flag files are listed in S2 Text.

III. References

[1] Gropp W, Lusk E, Doss N, Skjellum A. A high-performance, portable implementation of the MPI message passing interface standard. Parallel Computing. 1996;22(6): 789-828.
