# Supplementary material for: Rosetta:MSF: a modular framework for multi-state computational protein design
Source: PLoS Comput Biol. 2017 Jun 12;13(6):e1005600. doi: 10.1371/journal.pcbi.1005600 (PMC5484525; doi:10.1371/journal.pcbi.1005600)
Supplement: S2 Text — (PDF) [file pcbi.1005600.s002.pdf]

Rosetta:MSF: a modular framework for multi-state computational protein design

Patrick Löffler, Samuel Schmitz, Enrico Hupfeld, Reinhard Sterner, Rainer Merkl

Details of software validation, benchmark datasets and their compilation

The datasets used for benchmarking are deposited on our website:
https://bioinf.ur.de/downl/MSF_bench.tar.gz

The following text lists the protocol of the energy-minimization performed via relax, the composition of the design and repack shell, the parameters used for benchmarking and the setup of MD simulations.

I. Relax and design shell compositions

Structures were relaxed using a fast-relax protocol with backbone restraints, defined by the following flags:

```
-ignore_unrecognized_res
-relax:constrain_relax_to_start_coords
-relax:ramp_constraints false
-ex1
-ex2
-use_input_sc
-correct
-restore_pre_talaris_2013_behavior
-no_his_his_pairE
-no_optH false
-flip_HNQ
-nstruct 1
-relax:fast
-extra_res_fa ligand.params
```

For more information see: https://www.rosettacommons.org/docs/latest/rosetta_basics/preparation/preparing-structures

Composition of design and repack shells

The design shells used for recapitulation calculations on the NMR ensemble hIFABP were defined as given in Table A1.

Table A1: Design shell residues and repack-shell residues of the NMR ensemble hIFABP.

PDB ID	Design-shell residues	Repack-shell residues
2mji	14 17 21 23 24 27 28 31 58 70 72 75 76 78 91 93 102 104 117 119 124	8 11 13 15 16 18 20 22 25 26 30 32 33 36 38 49 51 53 59 60 62 68 69 71 73 74 77 79 80 82 89 90 92 94 95 96 100 101 103 105 106 115 116 118 122 123 126

Table A2 lists the design and repack shells used in *BR_EnzBench* and *MD_EnzBench*. Residues missing in the crystal structure were loop-modeled with YASARA, residues were renumbered to start with 1.

Table A2: Design shell and repack-shell residues of *BR_EnzBench* and *MD_EnzBench.*

PDB ID	Design-shell residues	Repack-shell residues
1fzq	23 25 26 27 28 29 30 31 32 33 66 90 92 125 126 128 129 158 159 160	21 22 24 34 35 51 53 64 65 67 88 91 93 94 95 102 123 124 127 130 131 132 156 157 161 162 163 164 165 177
1hsl	11 14 52 69 70 71 72 90 117 119 120 121 122 123 143 160 161 192 195	10 12 13 15 17 18 30 51 53 55 56 67 68 73 74 76 77 83 87 88 89 91 92 116 118 124 125 140 141 142 146 158 159 162 163 164 165 190 191 193 194 196 239
1j6z	8 10 11 12 13 15 30 71 151 153 154 155 178 179 180 182 183 207 210 211 214 298 299 300 302 303 333	9 14 24 27 28 29 66 68 69 70 103 105 106 134 138 152 156 157 158 181 184 185 186 203 204 206 208 209 212 213 215 216 254 255 258 297 301 304 306 332 334 335 336 337 338 370
1n4h	20 21 22 55 58 59 62 65 96 97 99 100 102 103 104 111 112 113 123 128 131 132 135 212 216	16 19 23 24 26 27 30 51 52 53 54 56 57 60 61 63 64 66 68 69 93 94 95 98 101 110 114 115 116 117 118 119 120 122 126 129 130 133 134 136 137 138 139 149 152 156 213 215 219 220 239 245
1nq7	20 21 22 55 58 59 62 65 93 96 97 99 100 102 103 111 112 113 123 126 128 131 132 135 136 212 213 216	16 17 18 19 23 24 26 27 43 51 52 53 54 56 57 60 61 63 64 66 68 69 92 94 95 98 101 104 110 114 115 117 118 119 120 121 122 124 129 130 133 134 138 139 149 152 156 209 210 211 214 215 217 219 220 239 243 245
1opb	20 25 29 33 36 38 40 42 51 53 55 57 58 59 60 62 76 77 106 108 117 119	4 8 10 13 16 17 19 21 22 23 30 31 32 34 35 37 39 41 44 49 50 52 54 56 61 63 64 72 74 75 78 84 86 93 94 95 97 104 105 107 109 110 115 116 118 120 121 126 128 130 134
1pot	9 10 11 12 37 58 107 143 145 146 186 187 204 206 207 230 232 268 302	8 13 33 34 35 36 38 39 40 56 57 59 60 61 104 105 106 108 109 141 142 144 147 149 150 185 188 189 190 191 202 203 205 210 228 229 231 233 266 301 303 323
1urg	43 44 45 64 66 67 112 145 148 149 150 151 206 224 226 257 259 327 337	8 9 10 38 42 47 62 63 65 68 70 71 97 109 110 111 113 114 144 147 153 205 222 223 225 227 254 255 256 258 260 295 296 328 329 333 334 336 338 340 374
2b3b	8 9 13 41 42 43 66 119 224 242 244 276 278 312 347 348 349	7 10 12 14 15 16 17 37 39 40 44 45 46 47 64 65 67 70 116 117 118 120 121 165 167 168 170 171 223 225 228 241 243 245 247 248 275 277 279 308 313 314 345 346 350 357 393
2dri	13 15 16 89 90 102 103 105 131 132 135 137 164 189 190 192 214 215 235	8 9 10 12 14 18 19 41 64 65 66 67 68 87 88 91 104 136 138 139 140 141 163 165 166 188 191 193 194 195 213 216 217 219 232 233 234 236 237 240 263 265 272
2ifb	17 18 23 27 30 31 38 49 51 60 62 70 72 73 74 78 82 91 93 102 104 117	2 6 11 14 15 19 20 21 22 24 26 28 29 32 34 36 39 40 47 48 50 53 54 55 56 58 59 61 63 68 69 71 75 76 77 79 80 81 89 90 92 94 95 103 105 106 115 116 118 119 122 124 125 126 132
2q2y	98 99 100 101 102 110 113 115 116 117 119 120 143 154 155 194 197 198 200 201 203 204 222	61 95 96 97 103 108 109 111 112 114 118 121 122 123 124 141 142 144 145 193 195 196 199 202 205 207 208 215 220 221 223 224 246 248 331
2qo4	17 18 21 23 27 30 31 34 36 51 53 54 55 56 72 73 74 75 76 98 111 118	8 11 13 14 15 16 20 22 24 28 29 32 33 35 37 38 49 52 57 58 60 70 71 77 78 91 93 95 96 97 99 100 109 110 112 113 116 119 120 122 126
2rct	20 24 29 33 37 40 42 44 55 57 59 61 62 63 64 66 80 81 110 112 121 123	8 12 14 17 21 23 25 26 27 28 30 34 35 36 38 39 41 43 45 46 48 53 54 56 58 60 65 67 68 76 78 79 82 88 90 97 98 99 101 108 109 111 113 119 120 122 124 125 130 132 134 142
2rde	73 75 79 112 113 114 115 117 139 140 141 143 144 145 146 184 185 196 197 198	17 18 20 71 72 74 76 77 78 80 81 110 111 116 118 119 120 137 138 142 147 148 158 179 181 182 183 186 187 194 195 199 204 207 211 225
2uyi	100 101 102 103 104 112 115 117 118 119 121 122 145 156 157 196 199 200 202 203 205 206 224	63 97 98 99 105 110 111 113 114 116 120 123 124 125 126 143 144 147 154 163 195 197 198 201 204 207 209 210 217 222 223 225 226 248 250 332

II. Parameters for *BR_EnzBench* and *hIFABP*

Below, flags are given for running `enzdes` on the first benchmark protein 1fzq of *BR_EnzBench*. Using the above given shell composition, the benchmark computations can be easily reproduced for other benchmark proteins and the hIFABP ensemble (2mji).

Parameter set for running enzdes:

1fzq.flags:

```
-in:file:l ./lists/1fzq_all # a list with paths to all conformations to design
-resfile ./resfiles/1fzq.resfile # using above defined design shell
-no_his_his_pairE
-correct
-restore_pre_talaris_2013_behavior
-extra_res_fa ./params/1fzq.params
# ENZDES flags
-enzdes::cst_design
-enzdes::design_min_cycles 2
-out::nstruct 1000
-enzdes::cst_min
-enzdes::chi_min
-enzdes::bb_min
-enzdes::lig_packer_weight 1.8
-enzdes::final_repack_without_ligand
-ex1
-ex2
-ex1aro
-ex2aro
-extrachi_cutoff 1
-soft_rep_design
-flip_HNQ
-linmem_ig 10
-docking::ligand::old_estat
-out:file:o ./output/1fzq_energy
-out:prefix ./output/1fzq_design
-run:constant_seed
-run:jran 11111111
```

./resfiles/1fzq.resfile:

```
NATRO
start
...
21 A NATAA  EX 1 EX 2 EX ARO 1 EX ARO 2  # repack shell
22 A NATAA  EX 1 EX 2 EX ARO 1 EX ARO 2
23 A ALLAA  EX 1 EX 2 EX ARO 1 EX ARO 2  # design shell
...
```

Parameter set for running `MSF:GA:enzdes`

In the following, example flags are given for running `MSF:GA:enzdes` on the first ensemble of benchmark protein 1fzq. Using the information of the above shell composition and modifying few flags, the benchmark computations can be easily reproduced. The way how an `MSF` run is set up follows the way implemented in `MPI_MSD` and thus, the definition of *.resfile files, *.daf files, *.state files, *.corr files, *.2resfile files for `MSF` is exactly the same.

See https://www.rosettacommons.org/docs/latest/application_documentation/design/mpi-msd for a more detailed documentation on how to prepare and customize a multi-state run.

1fzq.flags:

```
-entity_resfile ./resfiles/1fzq.resfile
-msf::fitness_file 1fzq.daf
-msf::pop_size 210
-msf::generations 600 # 800 for hIFABP
-msf::fraction_by_recombination 0.05
-msf::seed_sequences AAAAAAAAAAAAAAAAAAAA # length of DS - ALA seed
-msf::resfile_tmpdir ./tmp_resfiles/1fzq/ # temporary resfiles
-msf::checkpoint_write_interval 1
-msf::checkpoint_prefix ./checkpoints/1fzq/checkpoint
-no_his_his_pairE
-correct
-restore_pre_talaris_2013_behavior
-extra_res_fa ./params/1fzq.params
# ENZDES flags
-enzdes::cst_design
-enzdes::design_min_cycles 2
-enzdes::cst_min
-enzdes::chi_min
-enzdes::bb_min
-enzdes::lig_packer_weight 1.8
-enzdes::final_repack_without_ligand
-ex1
-ex2
-ex1aro
-ex2aro
-extrachi_cutoff 1
-soft_rep_design
-flip_HNQ
-linmem_ig 10
-docking::ligand::old_estat
-out:file:o msd_output/1fzq_energies
-out:prefix msd_output/1fzq_design
-run:constant_seed
-run:jran 11111111
```

./resfiles/1fzq.resfile:

```
20
ALLAA EX 1 EX 2 EX ARO 1 EX ARO 2
Start
```

1fzq.daf:

```
STATE_VECTOR state1 ./states/1fzq/state1
STATE_VECTOR state2 ./states/1fzq/state2
STATE_VECTOR state3 ./states/1fzq/state3
STATE_VECTOR state4 ./states/1fzq/state4
STATE_VECTOR state5 ./states/1fzq/state5
SCALAR_EXPRESSION best_state1 = vmin( state1 )
SCALAR_EXPRESSION best_state2 = vmin( state2 )
SCALAR_EXPRESSION best_state3 = vmin( state3 )
SCALAR_EXPRESSION best_state4 = vmin( state4 )
SCALAR_EXPRESSION best_state5 = vmin( state5 )
SCALAR_EXPRESSION best_sum = best_state1 + best_state2 + best_state3 + best_state4 +
   best_state5
FITNESS best_sum
```

./states/1fzq/state1:

```
./1fzq/input/1_backrub_input.pdb ./corr/1fzq.corr ./resfiles/1fzq.2resfile
```

./corr/1fzq.corr:

```
1 23 A
2 25 A
3 26 A
4 27 A
5 28 A
6 29 A
7 30 A
8 31 A
9 32 A
10 33 A
11 66 A
12 90 A
13 92 A
14 125 A
15 126 A
16 128 A
17 129 A
18 158 A
19 159 A
20 160 A
```

./resfiles/1fzq.2resfile:

```
NATRO
start
21 A NATAA EX 1 EX 2 EX ARO 1 EX ARO 2
22 A NATAA EX 1 EX 2 EX ARO 1 EX ARO 2
23 A NATAA EX 1 EX 2 EX ARO 1 EX ARO 2
24 A NATAA EX 1 EX 2 EX ARO 1 EX ARO 2
25 A NATAA EX 1 EX 2 EX ARO 1 EX ARO 2
26 A NATAA EX 1 EX 2 EX ARO 1 EX ARO 2
27 A NATAA EX 1 EX 2 EX ARO 1 EX ARO 2
28 A NATAA EX 1 EX 2 EX ARO 1 EX ARO 2
29 A NATAA EX 1 EX 2 EX ARO 1 EX ARO 2
30 A NATAA EX 1 EX 2 EX ARO 1 EX ARO 2
31 A NATAA EX 1 EX 2 EX ARO 1 EX ARO 2
32 A NATAA EX 1 EX 2 EX ARO 1 EX ARO 2
33 A NATAA EX 1 EX 2 EX ARO 1 EX ARO 2
34 A NATAA EX 1 EX 2 EX ARO 1 EX ARO 2
35 A NATAA EX 1 EX 2 EX ARO 1 EX ARO 2
51 A NATAA EX 1 EX 2 EX ARO 1 EX ARO 2
53 A NATAA EX 1 EX 2 EX ARO 1 EX ARO 2
64 A NATAA EX 1 EX 2 EX ARO 1 EX ARO 2
65 A NATAA EX 1 EX 2 EX ARO 1 EX ARO 2
66 A NATAA EX 1 EX 2 EX ARO 1 EX ARO 2
67 A NATAA EX 1 EX 2 EX ARO 1 EX ARO 2
88 A NATAA EX 1 EX 2 EX ARO 1 EX ARO 2
90 A NATAA EX 1 EX 2 EX ARO 1 EX ARO 2
91 A NATAA EX 1 EX 2 EX ARO 1 EX ARO 2
92 A NATAA EX 1 EX 2 EX ARO 1 EX ARO 2
93 A NATAA EX 1 EX 2 EX ARO 1 EX ARO 2
94 A NATAA EX 1 EX 2 EX ARO 1 EX ARO 2
95 A NATAA EX 1 EX 2 EX ARO 1 EX ARO 2
102 A NATAA EX 1 EX 2 EX ARO 1 EX ARO 2
123 A NATAA EX 1 EX 2 EX ARO 1 EX ARO 2
124 A NATAA EX 1 EX 2 EX ARO 1 EX ARO 2
125 A NATAA EX 1 EX 2 EX ARO 1 EX ARO 2
126 A NATAA EX 1 EX 2 EX ARO 1 EX ARO 2
127 A NATAA EX 1 EX 2 EX ARO 1 EX ARO 2
128 A NATAA EX 1 EX 2 EX ARO 1 EX ARO 2
129 A NATAA EX 1 EX 2 EX ARO 1 EX ARO 2
130 A NATAA EX 1 EX 2 EX ARO 1 EX ARO 2
131 A NATAA EX 1 EX 2 EX ARO 1 EX ARO 2
132 A NATAA EX 1 EX 2 EX ARO 1 EX ARO 2
156 A NATAA EX 1 EX 2 EX ARO 1 EX ARO 2
157 A NATAA EX 1 EX 2 EX ARO 1 EX ARO 2
158 A NATAA EX 1 EX 2 EX ARO 1 EX ARO 2
```

```
159 A NATAA EX 1 EX 2 EX ARO 1 EX ARO 2
160 A NATAA EX 1 EX 2 EX ARO 1 EX ARO 2
161 A NATAA EX 1 EX 2 EX ARO 1 EX ARO 2
162 A NATAA EX 1 EX 2 EX ARO 1 EX ARO 2
163 A NATAA EX 1 EX 2 EX ARO 1 EX ARO 2
164 A NATAA EX 1 EX 2 EX ARO 1 EX ARO 2
165 A NATAA EX 1 EX 2 EX ARO 1 EX ARO 2
177 B NATAA EX 1 EX 2 EX ARO 1 EX ARO 2
```

III. Parameters for *MD_EnzBench*

Preparation of structures and running MD simulations

All MD simulations were performed with YASARA Structure (version 14.7.17) employing the YAMBER3 [1] force field. Simulations were run at 298 K under periodic boundary conditions and with explicit water, using a multiple time step of 1 fs for intramolecular and 2 fs for intermolecular forces. Lennard Jones forces and long-range electrostatic interactions were treated with a 7.86 Å cutoff, the latter were calculated using the Particle Mesh Ewald method [2]. Temperature was adjusted using a Berendsen thermostat based on the time-averaged temperature and simulations were carried out at constant pressure. MD simulations require the definition of a simulation cell that should be adequately sized to prevent self-interaction through periodic boundaries. Simulation cells were thus defined as 5 Å larger than the protein along each axis. Cells were filled with water to a density of 0.997 g/ml, and counterions were added to a final concentration of 0.9% NaCl. Next, the protonation states of all molecules were assigned according to reference [3]. Usually, one removes conformational stress by means of an equilibration run prior to production runs. Here, we performed an energy minimization, which was done as follows: Following a steepest descent minimization, the procedure continued by simulated annealing (time step 2 fs, atom velocities scaled down by 0.9 every 10th step) until convergence was reached. The procedure was stopped, if the energy improved by less than 0.05 kJ/mol per atom during 200 steps.

Parameter set for running `enzdes` on *MD_EnzBench*

Flags for the computation of the single-state designability of *MD_EnzBench* are the same as those described in chapter II for `enzdes`, but "`-out::nstruct 1000`" was replaced with "`-out::nstruct 1`".

IV. Verification of **MSF:GA:AnchoredDesign**

To demonstrate the flexibility of MSF, we additionally integrated AnchoredDesign, which is used for anchored protein-protein interface design. This protocol redesigns the protein-protein interface of a scaffold protein by exploiting already known interactions (named anchor) of the target protein with a binding partner. The anchor is grafted into a surface loop of the scaffold and the interface loop (IL) that encloses the anchor is subsequently adapted via flexible redesign to generate a new binding partner. AnchoredDesign uses a two-step protocol. In the first phase, it performs a fast and coarse optimization utilizing Rosetta's centroid representation of atoms [4], which enables the protocol to consider large structural changes of ILs. In the second phase, the structure is refined by means of a full atom representation. The algorithm combines sequence design, extensive loop modeling, and rigid body docking, which is computationally demanding. Computing time is mostly determined by the length of the considered loop and the parameters that control the extent of IL structural sampling.

The dataset that was initially used to assess the performance of AnchoredDesign, is a structure prediction benchmark set whose sequences are fixed [5]. It was utilized to assess the performance of the algorithm in correctly predicting the structure of ILs given their native sequence and the position of the anchor residue. Although it allows one to demonstrate loop sampling capabilities of AnchoredDesign, this benchmark was not meaningful for assessing protein design protocols, which additionally requires the optimization of sequences.

Due to the high demand of computational resources of AnchoredDesign, we could assess only one example that combined loop sampling and sequence design. Thus, the results presented here are no comprehensive benchmark, but serve as a verification of MSF:GA:AnchoredDesign. We selected the first test case of the original benchmark, the factor B serine protease domain from *Homo sapiens* (PDB ID 1dle). This domain possesses an IL of eight residues and forms a homodimer. To create an ensemble, five unrelated conformations were generated by using the Backrub server [6]. Next, AnchoredDesign and MSF:GA:AnchoredDesign were used to compute the sequence and structure of the IL and sequence recovery was determined.

For SSD, 1000 randomly seeded runs of AnchoredDesign were performed using each of the five conformations and the parameter set *ps_anchored*; see the protocol below. For MSD, we first generated an initial population of sequences by means of coarse-grained sampling. Therefore, using the centroid- and MSF-specific parameter set *ps_anchored*, MSF:GA:AnchoredDesign was applied to the ensemble and executed for 1000 generations in centroid mode on a population of size 50 seeded with all-alanine sequences. The final population was the input of a second run, which was executed for 500 generations on a population consisting of 50 sequences based on the MSF-specific parameter set *ps_anchored*.

Similar to the assessment of enzdes (main text), *nsr*, *nssr* and *ts* values were determined: For SSD, these values were deduced for each conformation from the energetically best sequence in any of the 1000 runs. For MSD, these values were determined for the energetically top five sequences belonging to the final population of the refinement run. These values are given in Table A3. We confirmed convergence of both optimization algorithms by plotting the best *ts* values reached after *i* runs or *j* generations (Fig A1). In agreement with the enzdes protocol (main text), the sequence recovery of MSD is higher than that reached by the SSD protocol, as indicated by the *nsr* and *nssr* values.

Table A3: Sequence recovery and total score values for anchored protein-protein interface design of the factor B serine protease domain from *Homo sapiens* (PDB ID 1dle).

Protocol	*nsr* (%)	*nssr* (%)	*ts* (REU)
`AnchoredDesign`	40.00	51.42	-717.78
`MSF:GA:AnchoredDesign`	54.29	57.14	-699.00

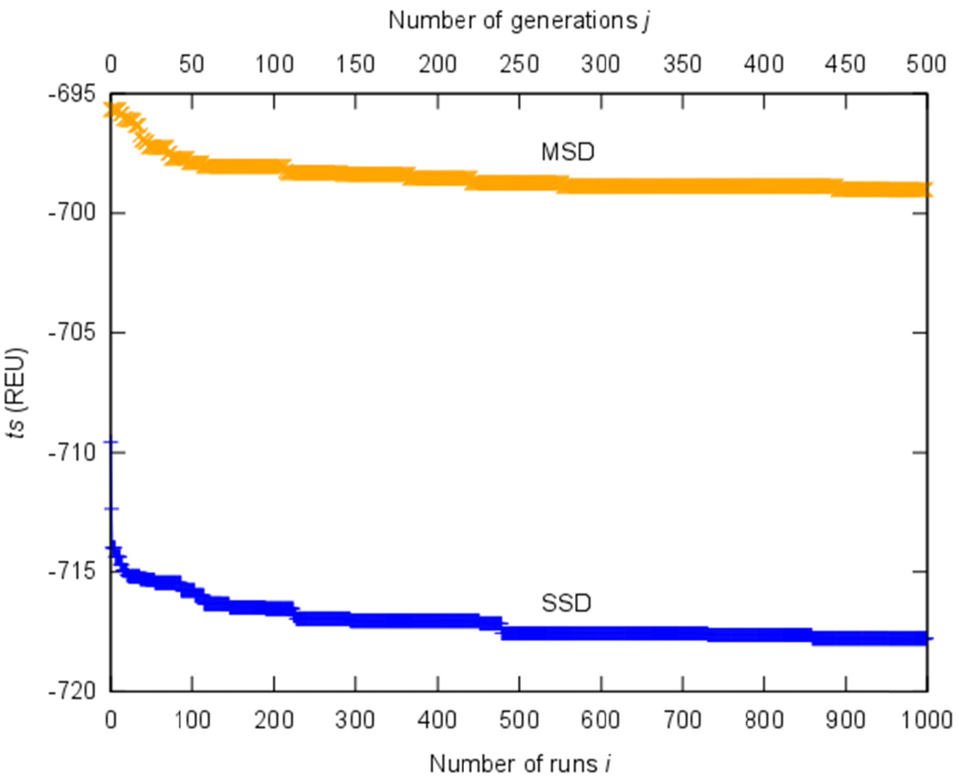

Fig A1: Convergence of SSD and MSD algorithms for anchored protein-protein interface design of the factor B serine protease domain (PDB ID 1dle).
The *ts*(*i*) and *ts*(*j*) values of `AnchoredDesign` (blue) and `MSF:GA:AnchoredDesign` (orange) are near to the optimum after 1000 runs or 500 generations of refinement, respectively.

Parameter set and design shell for 1dle

The protein was renumbered so that the first residue had residue number one. The design shell consisted of residues 27, 28, 29, 30, 31, 33, and 34; the anchor was residue 32, all were located on chain B.

Parameter set *ps_anchored*:

```
# protocol for coarse optimization
-unmute protocols.loops.CcdLoopClosureMover
#repeating options for safety
-run::version
-options::user
#packing options - these are about as high as they can go
-ex1
-ex2
-use_input_sc
-extrachi_cutoff 8
-linmem_ig 42
#minimization options
-run::min_type dfpmin_armijo
-nblist_autoupdate
#loops options
-loops::vicinity_sampling true
-loops::loop_file ../../loopsfile
#AnchoredDesign options
-AnchoredDesign
-anchor ../../anchor
-allow_anchor_repack false
-vary_cutpoints true
-debug false
-show_extended false
-refine_only false
-perturb_show false
-perturb_temp 0.8
-refine_temp 0.8
-refine_repack_cycles 50
-rmsd false
-unbound_mode false
-no_frags false
-perturb_CCD_off false
-perturb_KIC_off false
-refine_CCD_off false
-refine_KIC_off false
-chainbreak_weight 2.0
-testing::VDW_weight 2
#sample-size command
-AnchoredDesign::perturb_cycles 50
-AnchoredDesign::refine_cycles 100
-nstruct 1000
```

Changes for running MSF:

```
-nstruct 1 # change existing value to 1
# additional flags
-msf:fitness_file ../daf1 # fitness = sum of single states
-msf:entity_resfile ../entity_resfile
-msf:resfile_tmpdir ../tmp_resfiles1
-msf:checkpoint_prefix ../checkpoints1/checkpoint
-msf::pop_size 50
-msf::generations 1000
-msf::fraction_by_recombination 0.05
-msf::checkpoint_write_interval 1
-ignore_unrecognized_res
```

Changes for running MSF in centroid mode:

```
-AnchoredDesign::refine_cycles 0 # change existing value
```

V. Computing time

`Enzdes` protocol

In order to determine the computing time of single-state `enzdes` and multi-state `MSF:GA:enzdes`, exemplary calculations that used the above parameter sets were performed on eight cores of an Opteron K10 2354 for one Backrub conformation of the benchmark protein 1fzq. To process one conformation, 16 MC runs (`enzdes`) required 33 minutes and 16 GA generations with a population size of 210 sequences (`MSF:GA:enzdes`) required 295 minutes. The GA is implemented in an embarrassingly parallel fashion and scales linear with the number of states and the population size. This allows one to extrapolate the computing time for 1000 runs and 600 generations, which was sufficient to reach energetic convergence: Approximated for an ensemble of 20 conformations, `enzdes` demands a total computing time of 5500 core hours and `MSF:GA:enzdes` 29500 core hours. Thus, `MSF:GA:enzdes` required five times the computing time of `enzdes`.

`AnchoredDesign` protocol

To estimate the computational demand for anchored protein-protein interface design, computing times were measured for `AnchoredDesign` and `MSF:GA:AnchoredDesign` per run and generation, respectively. Calculations were performed on eight cores of an Opteron K10 2354 for protein 1dle and the above defined parameters. To process one conformation, 16 runs of `AnchoredDesign` required 243 minutes, while 16 generations with a population size of 50 sequences (`MSF:GA: AnchoredDesign`) took 195 minutes in centroid mode (first phase) and 2208 minutes in refinement mode (second phase). These numbers allow one to extrapolate the computing time for 1000 runs and 1000/500 generations in centroid/refinement mode: Approximated for an ensemble of 20 conformations and the given number of runs and generations, `AnchoredDesign` demands a total computing time of 40500 core hours and MSF:GA:AnchoredDesign 32500/184000 core hours, respectively. Similar to the situation of enzyme design, `MSF:GA:AnchoredDesign` required about five fold the computing time of `AnchoredDesign`.

Generally, `AnchoredDesign` is computationally more demanding than the `enzdes` protocol: the computing times are about seven fold higher, both for SSD and MSD. Moreover, the parameters that control the number of loop design cycles were set to a relatively low number allowing only limited loop movements. For a typical production run, the numbers of `perturb_cycles` and `refine_cycles` are set to values > 1000, allowing extensive loop movements but increase the computing time by a factor of 20.

VI. References

[1] Krieger E, Darden T, Nabuurs SB, Finkelstein A, Vriend G. Making optimal use of empirical energy functions: force-field parameterization in crystal space. Proteins. 2004;57(4):678-83.

[2] Essmann U, Perera L, Berkowitz ML, Darden T, Lee H, Pedersen LG. A smooth particle mesh Ewald method. The Journal of Chemical Physics. 1995 Nov 15;103(19):8577-93.

[3] Krieger E, Nielsen JE, Spronk CA, Vriend G. Fast empirical pK a prediction by Ewald summation. Journal of Molecular Graphics and Modelling. 2006 Dec 31;25(4):481-6.

[4] Rohl CA, Strauss CE, Misura KM, Baker D. Protein structure prediction using Rosetta. Methods in Enzymology. 2004;383:66-93.

[5] Lewis SM, Kuhlman BA. Anchored design of protein-protein interfaces. PLoS One. 2011;6(6):e20872.

[6] Lauck F, Smith CA, Friedland GF, Humphris EL, Kortemme T. RosettaBackrub-a web server for flexible backbone protein structure modeling and design. Nucleic Acids Res. 2010;38(Web Server issue):W569-75.
