# Supplementary material for: Rosetta:MSF: a modular framework for multi-state computational protein design
Source: PLoS Comput Biol. 2017 Jun 12;13(6):e1005600. doi: 10.1371/journal.pcbi.1005600 (PMC5484525; doi:10.1371/journal.pcbi.1005600)
Supplement: S3 Text — (PDF) [file pcbi.1005600.s003.pdf]

Rosetta:MSF: a modular framework for multi-state computational protein design

Patrick Löffler, Samuel Schmitz, Enrico Hupfeld, Reinhard Sterner, Rainer Merkl

Multi-state approach to design retro-aldolases

Chapter I complements the main text with figures and information about the *in silico* design. The *in silico* evaluation of retro-aldolases is documented in chapter II and the sequences of initial variants chosen for expression are listed. Chapter III describes the stabilization of initial variants by means of PROSS and the RA* dataset used for comparison with RA_MSD* variants is listed in chapter IV.

I. Multi-state design

Multi-state design was performed on the scaffold protein indole-3-glycerolphosphate synthase from *Sulfolobus solfataricus* (ssIGPS). First, the ligand was removed and conformations were sampled by means of MD simulations. To obtain representative conformations, the snapshots of the trajectory were clustered and in total, 12 conformations were picked from different clusters. `Rosetta:Match` was used to graft the transition state in those conformations and in the original crystal structure. Matched transition states (*mTS*) predicted as putatively weak binders were discarded. 23 ensembles of matched transition states (ens_{mTS}) having a specific catalytic triad (unique positions and amino acid identities) were chosen. Finally, enzymes were designed with `MSF:GA:enzdes`.

MD simulations of the scaffold protein

Three 10 ns MD simulations were applied to the scaffold ssIGPS without ligand, generating a snapshot every picosecond (10,000 snapshots per trajectory). MD simulations were performed with `YASARA Structure` (version 14.7.17) employing the YAMBER3 [1] force field. Simulations were run at 298 K under periodic boundary conditions and with explicit water, using a multiple time step of 1 fs for intramolecular and 2 fs for intermolecular forces. To perform three individual simulations, independent calculations were seeded by slightly changing the simulation temperature (+-0.01 K) which reassigns the initial atom velocities. Lennard Jones forces and long-range electrostatic interactions were treated with a 7.86 Å cutoff, the latter were calculated using the Particle Mesh Ewald method [2]. Temperature was adjusted using a Berendsen thermostat based on the time-averaged temperature and simulations were carried out at constant pressure. MD simulations require the definition of a simulation cell that should be adequately sized to prevent self-interaction through periodic boundaries. Thus, simulation cells were defined as 5 Å larger than the protein along each axis. Cells were filled with water to a density of 0.997 g/ml, and counterions were added to a final concentration of 0.9% NaCl. Next, the protonation states of all molecules were assigned according to reference [3]. Prior to production runs, an equilibration run is commonly performed to remove conformational stress. Thus, we performed an energy minimization as follows: After a steepest descent minimization phase, the procedure continued by simulated annealing (time step 2 fs, atom velocities scaled down by 0.9 every 10th step) until convergence was reached. Minimization was stopped if the energy improved by less than 0.05 kJ/mol per atom during 200 steps.

Selection of representative conformations and matching

All snapshots (3 x 10,000 in total) were clustered with `Durandal`, using *smart-mode* enabled and *semi-auto* [0.03 .. 0.20]. Four structures were picked from each largest cluster of the 3 trajectories

and the crystal structure of ssIGPS was utilized as a further conformation. Next, the theozyme was grafted onto these 13 conformations with `Rosetta:Match`. The matcher proposes a number of ligand positions located within a cavity of the scaffold. The cavities of all conformations were detected by means of the tool `Rosetta:gen_apo_grids` as described in Rosetta's documentation. The theozyme definition was derived from previous work [4] and is given by:

```
CST::BEGIN
TEMPLATE:: ATOM_MAP: 1 atom_name: C5 C4 C3
TEMPLATE:: ATOM_MAP: 1 residue3: MTD
TEMPLATE:: ATOM_MAP: 2 atom_type: Nlys ,
TEMPLATE:: ATOM_MAP: 2 residue1: K
CONSTRAINT:: distanceAB:    1.51 0.2 50.0   1  0
CONSTRAINT::    angle_A:  110.   5.0  0.0  60. 0
CONSTRAINT::    angle_B:  110.  10.0  0.0  60. 1
CONSTRAINT::  torsion_A: -120.  20.0  0.05 60. 0
CONSTRAINT:: torsion_AB:    0. 180.0  0.05 60. 3
CONSTRAINT::  torsion_B:    0. 180.0  0.00 60. 3
CST::END

CST::BEGIN
TEMPLATE:: ATOM_MAP: 1 atom_name: O2 C5 C6
TEMPLATE:: ATOM_MAP: 1 residue3: MTD
TEMPLATE:: ATOM_MAP: 2 atom_type: OOC ,
TEMPLATE:: ATOM_MAP: 2 residue1: DE
CONSTRAINT:: distanceAB:   3.0   0.3 10.0   0  1
CONSTRAINT::    angle_A: 125.0  20.0  0.0  60. 0
CONSTRAINT::    angle_B: 125.0  25.0  0.05 60. 0
CONSTRAINT::  torsion_A: -60.0  20.0  0.01 60. 0
CONSTRAINT:: torsion_AB:   0.0 180.0  0.0  60. 3
CONSTRAINT::  torsion_B: 180.0 180.0  0.0  60. 3
CST::END

CST::BEGIN
TEMPLATE:: ATOM_MAP: 1 atom_name: O2 C5 C6
TEMPLATE:: ATOM_MAP: 1 residue3: MTD
TEMPLATE:: ATOM_MAP: 2 atom_type: OH ,
TEMPLATE:: ATOM_MAP: 2 residue1: ST
CONSTRAINT:: distanceAB:   3.0   0.30 10.    0  0
CONSTRAINT::    angle_A: 125.0  20.0   0.0  60. 0
CONSTRAINT::    angle_B: 125.0  25.0   0.05 60. 0
CONSTRAINT::  torsion_A:  60.0  20.0   0.01 60. 0
CONSTRAINT:: torsion_AB:   0.0 180.0   0.0  60. 3
CONSTRAINT::  torsion_B: 180.0 180.0   0.0  60. 3
CST::END
```

`Rosetta:Match` was executed seven times with different seeds. For each uniquely specified catalytic triad (amino acid residues and positions), the resulting matched transition states (*mTS*) were collected, as described in methods. In total, 23 ensembles ens_{mTS} containing 4 - 13 conformations were chosen. In Fig B1, the ens_{mTS} of design RA_MSD2 is shown, which consists of 6 conformations (states for MSD).

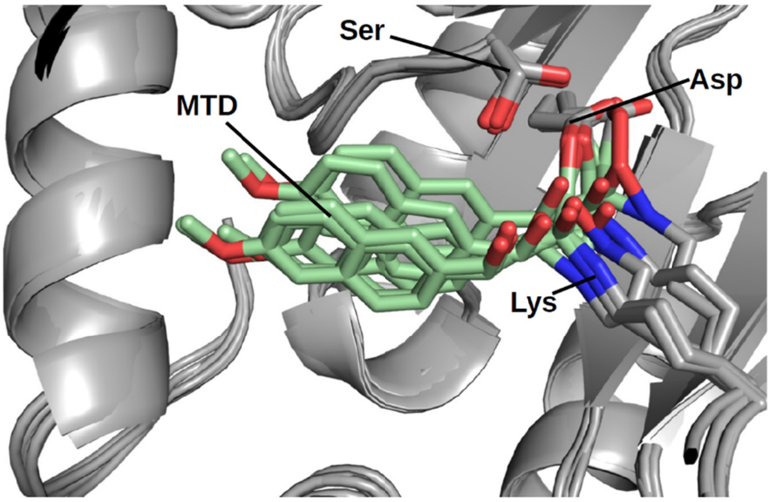

Fig B1. *ens$_{mTS}$* from design of RA_MSD2. Transition state of MTD (green) and the three catalytic residues (Lys, Asp, Ser) that were matched at positions 210, 131 and 110, respectively.

Multi-state design with **`MSF:GA:enzdes`**

All *ens$_{mTS}$* were designed with `MSF:GA:enzdes`; for the design of RA_MSD2 the following flags were used:

```
-entity_resfile ./corr_resfiles/RA_MSD2/entity_resfile
-msf::fitness_file ./daf/RA_MSD2.daf
-msf::pop_size 210
-msf::generations 2000
-msf::fraction_by_recombination 0.05
-msf::seed_sequence_using_correspondence_file ./corr_resfiles/RA_MSD2.corr
-msf::resfile_tmpdir tmp_resfiles/RA_MSD2/
-msf::checkpoint_write_interval 1
-msf::checkpoint_prefix checkpoints/RA_MSD2/checkpoint
-no_his_his_pairE
-correct
-restore_pre_talaris_2013_behavior
-extra_res_fa ./params/MTD_sb.params
-enzdes::cst_design
-enzdes::design_min_cycles 2
-enzdes::cst_min
-enzdes::chi_min
-enzdes::bb_min
-enzdes::favor_native_res 1.5
-ex1
-ex2
-ex1aro
-ex2aro
-use_input_sc
-extrachi_cutoff 1
-soft_rep_design
-flip_HNQ
-linmem_ig 10
-enzdes::lig_packer_weight 1.8
-docking::ligand::old_estat
-out:file:o ./energies/RA_MSD2/RA_MSD2_energies
-out:prefix ./output/RA_MSD2/
-enzdes::final_repack_without_ligand
-enzdes::cst_opt
-enzdes::cstfile ./cst/MTD.cst
-run:constant_seed
-run:jran 11111111
```

The `fitness_file` stores the fitness values determined as the sum of total energies over all states/conformations in ens_{mTS}. Designs were then run for 97 to 710 generations and Fig B2 shows the convergence for RA_MSD2:

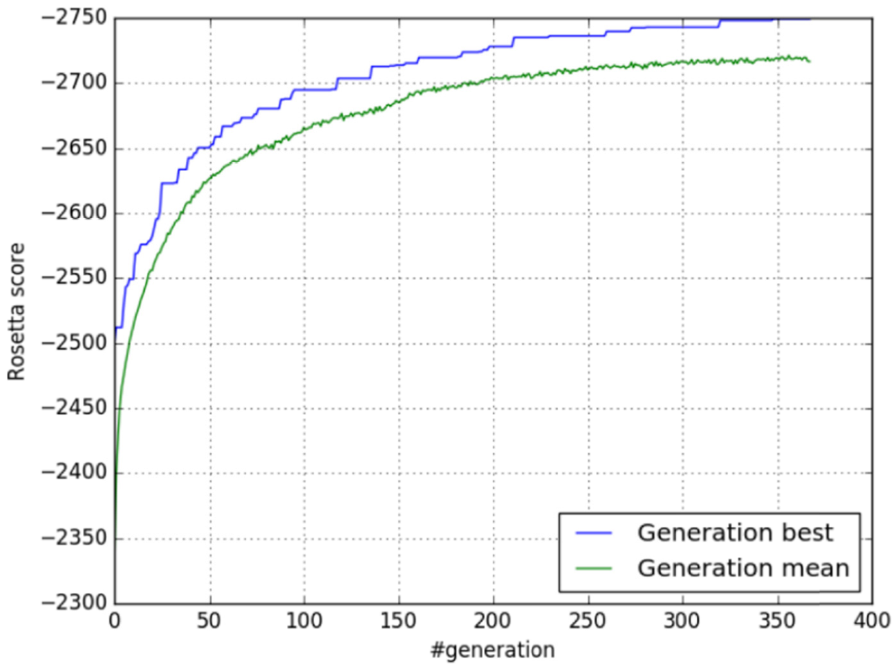

Fig B2. Energetic convergence of design run RA_MSD2: The number of generations used to design sequences plotted against their fitness (sum of Rosetta scores for all states). Lines show the fitness of each generation's best sequence (blue) and the mean fitness of the generation (green).

II. Evaluation of designs

For each design run, the 3D models related to the best scoring candidate sequences were visually inspected, followed by an assessment of Rosetta terms. It is difficult to compare scores of design runs originating from different conformations; thus we also assessed active-site geometries to rank the candidates. Next, the best 100 variants were further analyzed via MD simulations.

MD evaluation

As described in Materials and Methods, the best 100 designs were selected for 10 ns MD simulations in water and 100 snapshots were generated per simulation. Simulations were performed for (i) the enzyme/TS complex and (ii) the enzyme/substrate complex. Each trajectory was analyzed separately by determining for each snapshot the deviations from the distances and angles specified for the theozyme; see above definition. These data were plotted as scatter plots and as boxplots (Fig B3).

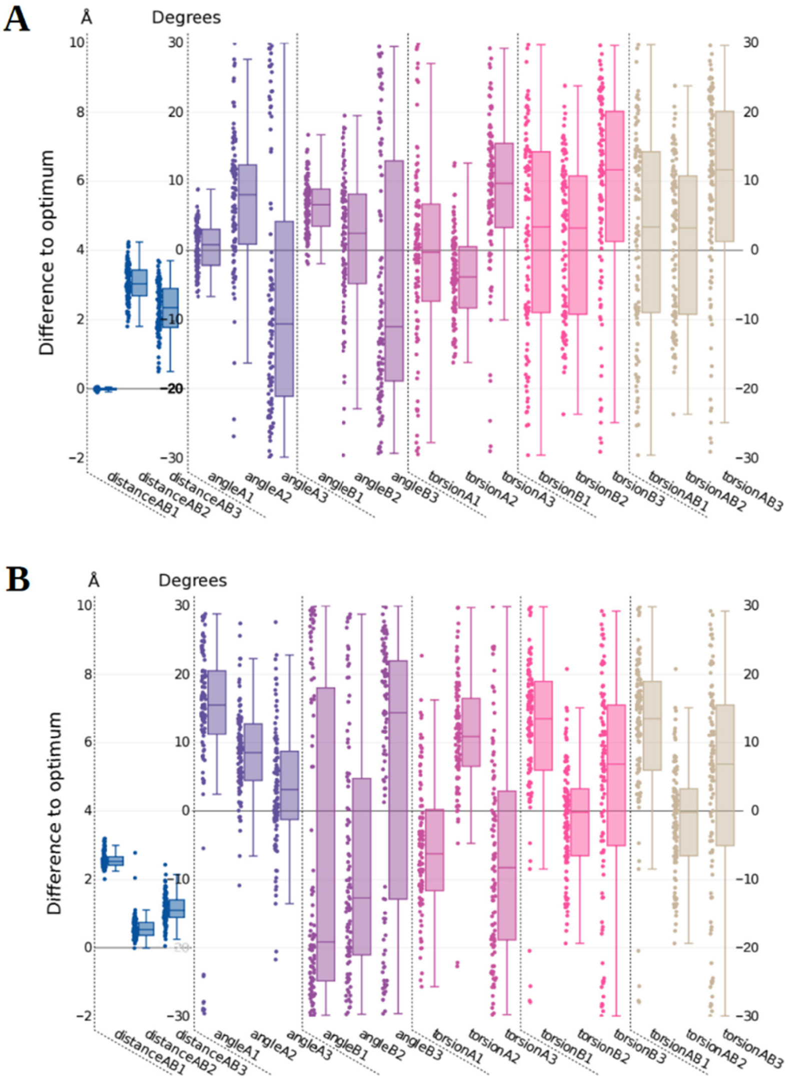

Fig B3. MD evaluation of RA_MSD2. The graphs show deviations of the optimal distances and angles specifying the theozyme. These values were deduced from 100 snapshots. Dots show the raw data, which were also plotted as boxplots. **A:** Deviations found in the enzyme/TS complex **B:** Deviations found in the enzyme/substrate complex.

Sequences of variants chosen for biochemical characterization

After filtering *in silico* for the best designs, the following sequences were chosen for biochemical characterization:

```
>RA_MSD1
MPRYLKGPMKRAVQLSLRRPSFRASRQRPIISLNERILEFNKRNITAIIAEYKRKEPSGLDVERDPIEYSKFMERYAVGLTIVTFEWYYNGSYE
TLRKIASSVSIPISMSDIIVKESQIDDAYNLGADTVDIIVKILTERELESLLEYARSYGMEPRIEINDENDLDIALRIGARFIVIDSRDPETLE
INKENQRKLISMIPSNVVKVAKSGISERNEIEELRKLGVNAFDIGSSLMRNPEKIKEFIL
>RA_MSD2
MPRYLKGPMKRAVQLSLRRPSFRASRQRPIISLNERILEFNKRNITAIIAEYKRKEPSGLDVERDPIEYSKFMERYAVGLTIVTFEKYYNGSYE
TLRKIASSVSIPISMSDVIVKESQIDDAYNLGADTVDLIVKILTERELESLLEYARSYGMEPRIDINDENDLDIALRIGARFICIDSRDPETLE
INKENQRKLISMIPSNVVKVAKSGISERNEIEELRKLGVNAFDIGSSLMRNPEKIKEFIL
>RA_MSD3
MPRYLKGPMKRAVQLSLRRPSFRASRQRPIISLNERILEFNKRNITAIIAEYKRKEPSGLDVERDPIEYSKFMERYAVGLTIVTFERYYNGSYE
TLRKIASSVSIPIAMSDIIVKESQIDDAYNLGADTVDIIVKILTERELESLLEYARSYGMEPRICINDENDLDIALRIGARFIAIDSRDPETLE
INKENQRKLISMIPSNVVKVAKSGISERNEIEELRKLGVNAFDIGSSLMRNPEKIKEFIL
>RA_MSD4
MPRYLKGWLKDVVQLSLRRPSFRASRQRPIISLNERILEFNKRNITAIIAKYERKHPSGLDVERDPIEYSKFMERYAVGLTISTLEKYFNGSYE
TLRKIASSVSIPIEMFDIIVKESQIDDAYNLGADTVVLVVKLLTERELESLLEYARSYGMEPLIIITDENDLDIALRIGARFIGIWSRDGETLE
INKENQRKLISMIPSNVVKVADGGISERNEIEELRKLGVNAFAIGESLMRNPEKIKEFIL
>RA_MSD5
MPRYLKGWLKDVVQLSLRRPSFRASRQRPIISLNERILEFNKRNITAIIAKYERKHPSGLDVERDPIEYSKFMERYAVGLMISTEEKYHNGSYE
TLRKIASSVSIPIAMFDIIVKESQIDDAYNLGADTVVLVVGLLTERELESLLEYARSYGMEPLIIITDENDLDIALRIGARFIGIWSRDGETLE
INKENQRKLISMIPSNVVKVAIGGISERNEIEELRKLGVNAFAIGESLMRNPEKIKEFIL
>RA_MSD6
MPRYLKGWVKDVVQLSLRRPSFRASRQRPIISLNERILEFNKRNITAIIAGYERKSLSGLDVERDPIEYSKFMERYAVGLFISTEEKYHNGSYE
TLRKIASSVSIPIGMVDGIVKESQIDDAYNLGADTVVLVVRLLTERELESLLEYARSYGMEPLIVIKDENDLDIALRIGARFIAIDSQDWETLE
INKENQRKLISMIPSNVVKVAVNGISERNEIEELRKLGVNAFKISASLMRNPEKIKEFIL
>RA_MSD7
MPRYLKGWLKDVVQLSLRRPSFRASRQRPIISLNERILEFNKRNITAIIALYGRKSPSGLDVERDPIEYSKFMERYAVGLAIFTEEKYHNGSYE
TLRKIASSVSIPICMTDFIVKESQIDDAYNLGADTVELYVKILTERELESLLEYARSYGMEPIITINDENDLDIALRIGARFIGILSRDLETLE
INKENQRKLISMIPSNVVKAAAEGISERNEIEELRKLGVNAFKIWESLMRNPEKIKEFIL
>RA_MSD8
MPRYLKGWLKDVVQLSLRRPSFRASRQRPIISLNERILEFNKRNITAIIAIYGRKSPSGLDVERDPIEYSKFMERYAVGLQIFTEEKYHNGSYE
TLRKIASSVSIPICMSDFIVKESQIDDAYNLGADTVELWVKILTERELESLLEYARSYGMEPIITINDENDLDIALRIGARFIGILSRDLETLE
INKENQRKLISMIPSNVVKAASEGISERNEIEELRKLGVNAFKIWESLMRNPEKIKEFIL
>RA_MSD9
MPRYLKGWMKDVVQLSLRRPSFRASRQRPIISLNERILEFNKRNITAIIAAYERKSPSGLDVERDPIEYSKFMERYAVGLSITTEEKYGNGSYE
TLRKIASSVSIPIDMTDVIVKESQIDDAYNLGADTVTLVVRILTERELESLLEYARSYGMEPLIVISDENDLDIALRIGARFICIDSRDWETLE
INKENQRKLISMIPSNVVKVAANGISERNEIEELRKLGVNAFKIGSSLMRNPEKIKEFIL
```

III. PROSS stabilization

Variant RA_MSD2 had the lowest activity of all designs and was insoluble on expression without MBP. To assess the relationship between activity and solubility, we predicted stabilizing mutations utilizing the PROSS server as described in Materials and Methods. Each sequence logo shown in Fig B4 was computed from an MSA that was generated by predicting seven stabilized variants for each conformational state of RA_MSD2 and by merging the predicted mutations for each degree of stabilization. According to PROSS, sequence logo 7 corresponds to the strongest stabilization.

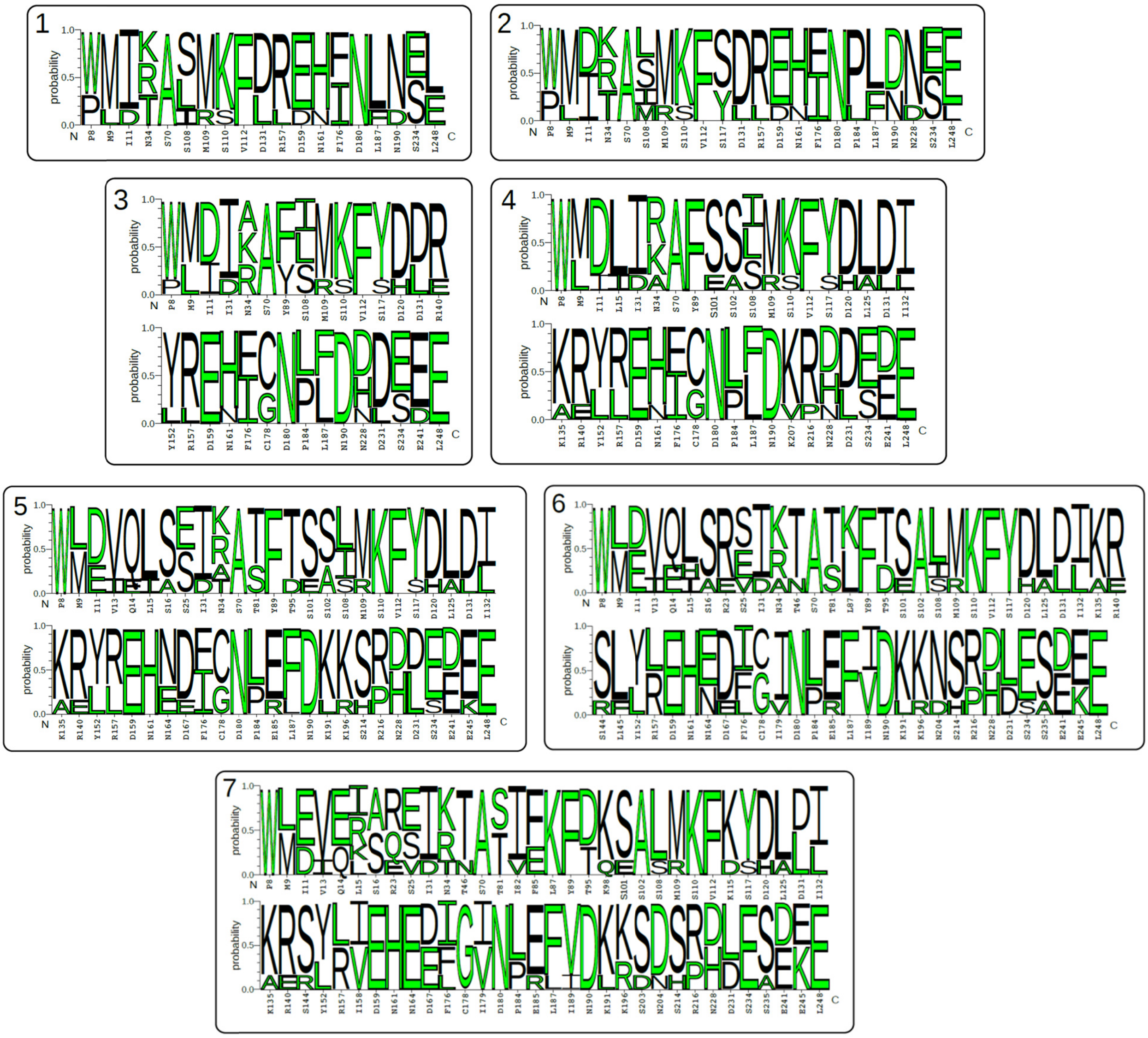

Fig B4. Sequence logos 1-7 resulting from PROSS predictions with an increasing number of putatively stabilizing mutations. Sequence logos were determined using weblogo. Black letters correspond to the native sequence and green letters indicate stabilizing mutations. The height of the letter represents the consensus from all designed conformational states.

Mutations proposed by PROSS that were selected for biochemical characterization

Based on the sequence logos, five sets of mutations were introduced in RA_ MSD2; compare Table 1.

Table B1. Mutations introduced into RA_MSD2 for stabilization. Positions in bold indicate additional mutations introduced with this variant. The last column contains the number of exchanges relative to the wildtype sequence.

Name	Amino acid exchanges compared to RA_MSD2	Number of exchanges compared to sequence of ssIGPS
RA_MSD2.1	**N34K S102A S117Y N164E N190D N204D N228H L248E** (8)	28
RA_MSD2.2	**S70A N161H C178G D180N I189V S234E** (6)	22
RA_MSD2.3	N34K S70A S102A S117Y N161H N164E I189V N190D N204D N228H S234E L248E (12)	32
RA_MSD2.4	**P8W M9L** N34K S70A **Y89F** S102A **S108L** S117Y N161H N164E C178G N190D N204D N228H S234E L248E (16)	26
RA_MSD2.5	P8W M9L **Q14E L15I S25E** N34K S70A Y89F S102A S108L S117Y N161H N164E C178G D180N I189V N190D N204D N228H S234E L248E (21)	29

Fig A5. Location of mutations. To generate variant RA_MSD2.1, surface positions (green spheres) were chosen and for RA_MSD2.2, core positions (yellow spheres) were selected. RA_MSD2.3 is a combination of mutations from RA_MSD2.1 and RA_MSD2.2. For variants RA_MSD2.4, additional core positions (orange spheres) and for RA_MSD2.5, additional surface positions (red spheres) were selected.

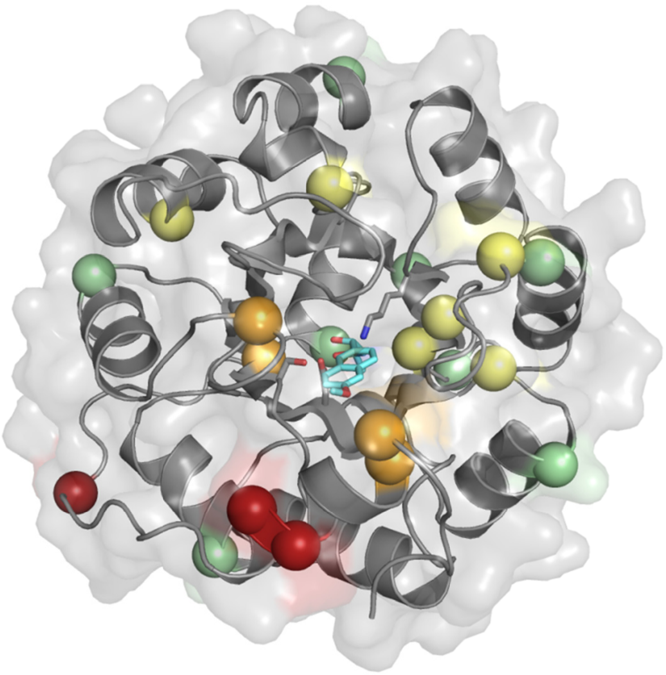

Sequences of stabilized variants chosen for expression

```
>RA_MSD2.1
MPRYLKGPMKRAVQLSLRRPSFRASRQRPIISLKERILEFNKRNITAIIAEYKRKEPSGLDVERDPIEYSKFMERYAVGLTIVTFEKYYNGSYE
TLRKIASAVSIPISMSDVIVKEYQIDDAYNLGADTVDLIVKILTERELESLLEYARSYGMEPRIDINDEEDLDIALRIGARFICIDSRDPETLE
IDKENQRKLISMIPSDVVKVAKSGISERNEIEELRKLGVHAFDIGSSLMRNPEKIKEFIE
>RA_MSD2.2
MPRYLKGPMKRAVQLSLRRPSFRASRQRPIISLNERILEFNKRNITAIIAEYKRKEPSGLDVERDPIEYAKFMERYAVGLTIVTFEKYYNGSYE
TLRKIASSVSIPISMSDVIVKESQIDDAYNLGADTVDLIVKILTERELESLLEYARSYGMEPRIDIHDENDLDIALRIGARFIGINSRDPETLE
VNKENQRKLISMIPSNVVKVAKSGISERNEIEELRKLGVNAFDIGESLMRNPEKIKEFIL
>RA_MSD2.3
MPRYLKGPMKRAVQLSLRRPSFRASRQRPIISLKERILEFNKRNITAIIAEYKRKEPSGLDVERDPIEYAKFMERYAVGLTIVTFEKYYNGSYE
TLRKIASAVSIPISMSDVIVKEYQIDDAYNLGADTVDLIVKILTERELESLLEYARSYGMEPRIDIHDEEDLDIALRIGARFICIDSRDPETLE
VDKENQRKLISMIPSDVVKVAKSGISERNEIEELRKLGVHAFDIGESLMRNPEKIKEFIE
>RA_MSD2.4
MPRYLKGWLKRAVQLSLRRPSFRASRQRPIISLKERILEFNKRNITAIIAEYKRKEPSGLDVERDPIEYAKFMERYAVGLTIVTFEKYFNGSYE
TLRKIASAVSIPILMSDVIVKEYQIDDAYNLGADTVDLIVKILTERELESLLEYARSYGMEPRIDIHDEEDLDIALRIGARFIGIDSRDPETLE
IDKENQRKLISMIPSDVVKVAKSGISERNEIEELRKLGVHAFDIGESLMRNPEKIKEFIE
>RA_MSD2.5
MPRYLKGWLKRAVEISLRRPSFRAERQRPIISLKERILEFNKRNITAIIAEYKRKEPSGLDVERDPIEYAKFMERYAVGLTIVTFEKYFNGSYE
TLRKIASAVSIPILMSDVIVKEYQIDDAYNLGADTVDLIVKILTERELESLLEYARSYGMEPRIDIHDEEDLDIALRIGARFIGINSRDPETLE
VDKENQRKLISMIPSDVVKVAKSGISERNEIEELRKLGVHAFDIGESLMRNPEKIKEFIE
```

IV. List of retro-aldolase sequences (RA*) used for comparison with multi-state variants (RA_MSD*)

For an easier comparison, termini were specified as `N-MPRYL…` and `…PEKIKE-C`. At the C-terminus, RA variants contain more amino acid and a his-tag to facilitate protein synthesis and purification. For example, the C-terminus of RA114.3 is `PEKIKELIEGSLEHHHHHH`.

```
>RA41
MPRYLKGWAKDVVQLSLRRPSFRASRQRPIISLNERILEFNKRNITAIIAVYSRKSPSGLDVERDPIEYSKFMERYAVGLTIYTEEKYWNGSYE
TLRKIASSVSIPILMADLIVKESQIDDAYNLGADTVVLIVPILTERELESLLEYARSYGMEPLIVIVDENDLDIALRIGARFIKIKSRDWETLE
INKENQRKLISMIPSNVVKVASSGISERNEIEELRKLGVNAFIIGSSLMRNPEKIKE
>RA115
MPRYLKGTLEDVVQLSLRRPSVRASRQRPIISLNERILEFNKRNITAIIASYTRKEPSGLDVERDPIEYAKFMERYAVGLSILTEEKWSNGSYE
TLRKIASSVSIPILMKDFIVKESQIDDAYNLGADTVLLIVKILTERELESLLEYARSYGMEPLIEINDENDLDIALRIGARFIGINSRDRETWE
INKENQRKLISMIPSNVVKVAEKGISERNEIEELRKLGVNAFLISSSLMRNPEKIKE
>RA114
MPRYLKGWLEDVVQLSLRRPSVRASRQRPIISLNERILEFNKRNITAIIAEYKRKDPSGLDVERDPIEYAKFMERYAVGLSILTEEKYFNGSYE
TLRKIASSVSIPILMSDFIVKESQIDDAYNLGADTVALIVKILTERELESLLEYARSYGMEPLIIINDENDLDIALRIGARFIGIASRDWETGE
INKENQRKLISMIPSNVVKVAKEGISERNEIEELRKLGVNAFEIGSSLMRNPEKIKE
>RA116
MPRYLKGWLEDVVQLSLRRPSVRASRQRPIISLNERILEFNKRNITAIIAGYSRKSPSGLDVERDPIEYAKFMERYAVGLSIMTEEKYFNGSYE
TLRKIASSVSIPIMMLDFIVKESQIDDAYNLGADTVLLLVKILTERELESLLEYARSYGMEPLIAINDENDLDIALRIGARFIGIYSRDPETLE
INKENQRKLISMIPSNVVKVAIGGISERNEIEELRKLGVNAFKIESSLNRNPEKIKE
>RA117
MPRYLKGWLEDVVQLSLRRPSVRASRQRPIISLNERILEFNKRNITAIIAEYKRKSPSGLDVERDPIEYAKFMERYAVGLKILTEEKYFNGSYE
TLRKIASSVSIPIAMSDVIVKESQIDDAYNLGADTVVLIVKLLTERELESLLEYARSYGMEPLIVINDENDLDIALRIGARFIGISSRDWETLE
INKENQRKLISMIPSNVVKVAISGISERNEIEELRKLGVNAFLIGSSLMRNPEKIKE
>RA118
MPRYLKGWLEDVVQLSLRRPSVRASRQRPIISLNERILEFNKRNITAIIAGYHRKDPSGLDVERDPIEYAKFMERYAVGLAIATEEKYANGSYE
TLRKIASSVSIPIEMWDFIVKESQIDDAYNLGADTVCLIVKILTERELESLLEYARSYGMEPLIKINDENDLDIALRIGARFIGIVSRDFETLE
INKENQRKLISMIPSNVVKVASFGISERNEIEELRKLGVNAFSILSSLMRNPEKIKE
>RA119
MPRYLKGWLEDAVQLSLRRPSVRASRQRPIISLNERILEFNKRNITAIIALYMRKMDAGLDVERDPIEYAKFMERYAVGLSILTSEKNHNGSYE
TLRKIASSVSIPILMWDMIVKESQIDDAYNLGADTVLLIVKILTERELESLLEYARSYGMEPLIKINDENDLDIALRIGARFIGISSEDSETLE
INKENQRKLISMIPSNVVKVAQSGISERNEIEELRKLGVNAFLIGSSLMRNPEKIKE
>RA120
MPRYLKGWLEDAVQLSLRRPSVRASRQRPIISLNERILEFNKRNITAIIAEYKRKSPSGLDVERDPIEYAKFMERYAVGLSILTSEKYFNGSYE
TLRKIASSVSIPIMMKDMIVKESQIDDAYNLGADTVKLTVKILTERELESLLEYARSYGMEPLIEINDENDLDIALRIGARFIGINSRDSETLE
INKENQRKLISMIPSNVVKVAQSGISERNEIEELRKLGVNAFLIGSSLMRNPEKIKE
>RA95.5-8
MPRYLKGWLEDVVQLSLRRPSVHASRQRPIISLNERILEFNKRNITAIIAYYLRKSPSGLDVERDPIEYAKYMERYAVGLSIKTEEKYFNGSYE
MLRKIASSVSIPILMNDFIVKESQIDDAYNLGADTVLLIVNILTERELESLLEYARSYGMEPLILINDENDLDIALRIGARFIVIFSMNFETGE
INKENQRKLISMIPSNVVKVAHLDISERNEIEELRKLGVNAFLISSSLMRNPEKIKE
>RA95.5-8F
MPRYLKGWLEDVVQLSLRRPSVHASRQRPIISLNERILEFNKRNITAIIAYYLRKSPSGLDVERDPIEYAKYMEPYAVGLSIKTEEKYFDGSYE
MLRKIASSVSIPILMNDFIVKESQIDDAYNLGADTVLLIVEILTERELESLLEYARGYGMEPLILINDENDLDIALRIGARFITIYSMNFETGE
INKENQRKLISMIPSNVVKVPLLDFFEPNEIEELRKLGVNAFMISSSLMRNPEKIKE
>RA114.3
```

MPRYLKGWLEDVVQLSLRRPSVRASRQRPIISLNERILEFNKRNITAIIAEYKRKDPSGLDVERDPIEYAKFMERYAVGLFISTEEKYFNGSYE
TLRKIASSVSIPILMYDFIVKESQIDDAYNLGADTVALIVKILTERELESLLEYARSYGMEPLIIINDENDLDIALRIGARFIGIAARDWETGE
INKENQRKLISMIPSNVVKVAKEGISERNEIEELRKLGVNAFLIGSSLMRNPEKIKE
>RA95.0
MPRYLKGWLEDVVQLSLRRPSVRASRQRPIISLNERILEFNKRNITAIIAVYERKSPSGLDVERDPIEYAKFMERYAVGLSITTEEKYFNGSYE
TLRKIASSVSIPILMSDFIVKESQIDDAYNLGADTVLLIVKILTERELESLLEYARSYGMEPLILINDENDLDIALRIGARFIGIMSRDFETGE
INKENQRKLISMIPSNVVKVAKLGISERNEIEELRKLGVNAFLISSSLMRNPEKIKE
>RA95.5-5
MPRYLKGWLEDVVQLSLRRPSVHASRQRPIISLNERILEFNKSNITAIIAYYTRKSPSGLDVERDPIEYAKFMERYAVGLSIKTEEKYFNGSYE
MLRKIASSVSIPILMNDFIVKESQIDDAYNLGADTVLLIVKILTERELESLLEYARSYGMEPLILINDENDLDIALRIGARFISIFSMNFETGE
INKENQRKLISMIPSNVVKVAKLGISERNEIEELRKLGVNAFLISSSLMRNPEKIKE
>RA95.5
MPRYLKGWLEDVVQLSLRRPSVRASRQRPIISLNERILEFNKRNITAIIAYYSRKSPSGLDVERDPIEYAKFMERYAVGLSIKTEEKYFNGSYE
TLRKIASSVSIPILMSDFIVKESQIDDAYNLGADTVLLIVKILTERELESLLEYARSYGMEPLILINDENDLDIALRIGARFIGIFSMNFETGE
INKENQRKLISMIPSNVVKVAKLGISERNEIEELRKLGVNAFLISSSLMRNPEKIKE
>RA117.1
MPRYLKGWLEDVVQLSLRRPSVRASRQRPIISLNERILEFNKRNITAIIAEYKRKSPSGLDVERDPIEYAKFMERYAVGLKILTEEKYFNGSYE
TLRKIASSVSIPIAMSDAIVKESQIDDAYNLGADTVVLIVKILTERELESLLEYARSYGMEPLIVINDENDLDIALRIGARFIGIESRDWETLE
INKENQRKLISMIPSNVVKVAIAGISERNEIEELRKLGVNAFLIGSSLMRNPEKIKE
>RA114.4
MPRYLKGWLEDVVQLSLRRPSVRASRQRPIISLNERILEFNKRNITAIIAEYKRKDPSGLDVERDPIEYAKFMERYAVGLFISTEEKYFNGSYE
TLRKIASSVSIPILMYDFIVKESQIDDAYNLGADTVALIVKILTERELESLLEYARSYGMEPLIIINDENDLDIALRIGARFIGIAARDWETGE
INKENQRKLISMIPSNVVKVAKEGISERNEIEELRKLGVNAFVTASGSLMRNPEKIKE
>RA22
MPRYLKGWLKDVVQLSLRRPSFRASRQRPIISLNERILEFNKRNITAIIAGYDRKSPSGLDVERDPIEYSKFMERYAVGLSITTEEKYFNGSYE
TLRKIASSVSIPILMADFIVKESQIDDAYNLGADTVALIVKILTERELESLLEYARSYGMEPLIKINDENDLDIALRIGARFIGIVSADWETLE
INKENQRKLISMIPSNVVKVAAFGISERNEIEELRKLGVNAFSIHSSLMRNPEKIKE
>RA34.6
MPRYLKGWLEDVVQLSLRRPSVRASRQRPIISLNERILEFNKRNITAIIATYMRKSPWGLDVERDPIEYAKFMERYAVGLSICTEEKYANGSYE
TLRKIASSVSIPILMADFIVKESQIDDAYNLGADTVPLIVKILTERELESLLEYARSYGMEPIIKINDENDLDIALRIGARFIGICSRDWETLE
INKENQRKLISMIPSNVVKVASTGISERNEIEELRKLGVNAFSIISSLMRNPEKIKE
>RA67
MPRYLKGWLKDVVQLSLRRPSFRASRQRPIISLNERILEFNKRNITAIIAKYKRKHPSGLDVERDPIEYSKFMERYAVGLSIWTEEKYFNGSYE
TLRKIASSVSIPILMSDFIVKESQIDDAYNLGADTVVLYVKILTERELESLLEYARSYGMEPLIVINDENDLDIALRIGARFIEIVSRDLETGE
INKENQRKLISMIPSNVVKVASSGISERNEIEELRKLGVNAFSIGSSLMRNPEKIKE
>RA90
MPRYLKGSLKDVVQLSLRRPSFRASRQRPIISLNERILEFNKRNITAIIAEYSRKSPWGLDVERDPIEYSKFMERYAVGLTILTEEKYFNGSYE
TLRKIASSVSIPILMSDVIVKESQIDDAYNLGADTVKLIVKILTERELESLLEYARSYGMEPLIVINDENDLDIALRIGARFIGILSRDLETLE
INKENQRKLISMIPSNVVKVASSGISERNEIEELRKLGVNAFLIGSSLMRNPEKIKE
>RA92
MPRYLKGSLKDVVQLSLRRPSFRASRQRPIISLNERILEFNKRNITAIIAEYTRKHPSGLDVERDPIEYSKFMERYAVGLSILTEEKYLNGSYE
TLRKIASSVSIPILMVDLIVKESQIDDAYNLGADTVVLIVKILTERELESLLEYARSYGMEPLIVINDENDLDIALRIGARFIGIKSRDFETLE
INKENQRKLISMIPSNVVKVALSGISERNEIEELRKLGVNAFLITSSLMRNPEKIKE
>RA98
MPRYLKGSLKDVVQLSLRRPSFRASRQRPIISLNERILEFNKRNITAIIAKYLRKSPWGLDVERDPIEYSKFMERYAVGLSILTEEKYTNGSYE
TLRKIASSVSIPILMVDFIVKESQIDDAYNLGADTVLLIVKILTERELESLLEYARSYGMEPLIEINDENDLDIALRIGARFILINSRDHETLE
INKENQRKLISMIPSNVVKVASSGISERNEIEELRKLGVNAFLIGSSLMRNPEKIKE
>RA68
MPRYLKGWLKDVVQLSLRRPSFRASRQRPIISLNERILEFNKRNITAIIAKYKRKSPTGLDVERDPIEYSKFMERYAVGLSISTEEKYHNGSYE
TLRKIASSVSIPILMMDFIVKESQIDDAYNLGADTVLLIVKILTERELESLLEYARSYGMEPLILINDENDLDIALRIGARFIGINSRDYETGE
TNKENQRKLISMIPSNVVKVAIYGISERNEIEELRKLGVNAFLISSSLMRNPEKIKE
>RA53
MPRYLKGWLKDVVQLSLRRPSFRASRQRPIISLNERILEFNKRNITAIIAVYSRKHPSGLDVERDPIEYSKFMERYAVGLSIYTEEKYTNGSYE
TLRKIASSVSIPILMVDVIVKESQIDDAYNLGADTVVLIVKILTERELESLLEYARSYGMEPLIKINDENDLDIALRIGARFIVISSYDWETLE
INKENQRKLISMIPSNVVKVASGGISERNEIEELRKLGVNAFSIGSSLMRNPEKIKE
>RA43
MPRYLKGWLKDVVQLSLRRPSFRASRQRPIISLNERILEFNKRNITAIIAVYSRKSPSGLDVERDPIEYSKFMERYAVGLLIWTGEKYGNGSYE
TLRKIASSVSIPILMVDWIVKESQIDDAYNLGADTVLLVVKILTERELESLLEYARSYGMEPLISIYDENDLDIALRIGARFIKIASRDPETLE
INKENQRKLISMIPSNVVKVASSGISERNEIEELRKLGVNAFVIGSSLMRNPEKIKE
>RA40
MPRYLKGWVKDVVQLSLRRPSFRASRQRPIISLNERILEFNKRNITAIIAVYMRKSPSGLDVERDPIEYSKFMERYAVGLTIYTEEKYFNGSYE
TLRKIASSVSIPILMVDFIVKESQIDDAYNLGADTVVLFVPILTERELESLLEYARSYGMEPLIVINDENDLDIALRIGARFIKILSSDVETLE
INKENQRKLISMIPSNVVKVASHGISERNEIEELRKLGVNAFSIGSSLMRNPEKIKE
>RA26
MPRYLKGWLKDVVQLSLRRPSFRASRQRPIISLNERILEFNKRNITAIIAEYSRKSPWGLDVERDPIEYSKFMERYAVGLLILTEEKYFNGSYE
TLRKIASSVSIPILMHDFIVKESQIDDAYNLGADTVKLIVKILTERELESLLEYARSYGMEPLIAIHDENDLDIALRIGARFIGISSRDPETLE
INKENQRKLISMIPSNVVKVALSGISERNEIEELRKLGVNAFLIGSSLMRNPEKIKE
>RA63
MPRYLKGWLKDVVQLSLRRPSFRASRQRPIISLNERILEFNKRNITAIIALYMRKSPWGLDVERDPIEYSKFMERYAVGLSILTEEKYFNGSYE
TLRKIASSVSIPILMHDFIVKESQIDDAYNLGADTVKLSVYILTERELESLLEYARSYGMEPLISINDENDLDIALRIGARFIGIVSRDPETLE
INKENQRKLISMIPSNVVKVAISGISERNEIEELRKLGVNAFLIGSSLMRNPEKIKE
>RA57
MFRYLKGWLKDVVQLSLRRPSFRASRQRPIISLNERILEFNKRNITAIIAGYMRKSPSGLDVERDPIEYSKFMERYAVGLSIWTEEKYSNGSYE
TLRKIASSVSIPILMLDFIVKESQIDDAYNLGADTVVLIVKILTERELESLLEYARSYGMEPLIKINDENDLDIALRIGARFIGIVSRDWETLE
INKENQRKLISMIPSNVVKVASHGISERNEIEELRKLGVNAFTIYSSLMRNPEKIKE

>RA56
MPRYLKGRLKDVVQLSLRRPSFRASRQRPIISLNERILEFNKRNITAIIAGYIRKHPSGLDVERDPIEYSKFMERYAVGLAIYTEEKYTNGSYE
TLRKIASSVSIPILMIDFIVKESQIDDAYNLGADTVVLIVKILTERELESLLEYARSYGMEPLIKINDENDLDIALRIGARFIGIHSRDWETFE
INKENQRKLISMIPSNVVKVATSGISERNEIEELRKLGVNAFSIYSSLMRNPEKIKE
>RA55
MPRYLKGWLKDVVQLSLRRPSFRASRQRPIISLNERILEFNKRNITAIIAYYTRKSPWGLDVERDPIEYSKFMERYAVGLSILTEEKYFNGSYE
TLRKIASSVSIPILMTDFIVKESQIDDAYNLGADTVALIVKILTERELESLLEYARSYGMEPLIKINDENDLDIALRIGARFIGIVSRDWETLE
INKENQRKLISMIPSNVVKVASSGISERNEIEELRKLGVNAFSIVISLMRNPEKIKE
>RA49
MPRYLKGWLKDVVQLSLRRPSFRASRQRPIISLNERILEFNKRNITAIIAMYSRKSPWGLDVERDPIEYSKFMERYAVGLVILTGEKYANGSYE
TLRKIASSVSIPILMVDWIVKESQIDDAYNLGADTVVLHVKILTERELESLLEYARSYGMEPLITINDENDLDIALRIGARFIKISSRDHETLE
INKENQRKLISMIPSNVVKVAALGISERNEIEELRKLGVNAFIIGSSLMRNPEKIKE
>RA48
MPRYLKGWLKDVVQLSLRRPSFRASRQRPIISLNERILEFNKRNITAIIAMYSRKSPLGLDVERDPIEYSKFMERYAVGLAIFTEEKYWNGSYE
TLRKIASSVSIPILMLDFIVKESQIDDAYNLGADTVKLSVLILTERELESLLEYARSYGMEPLISIYDENDLDIALRIGARFILIVSRDPETLE
INKENQRKLISMIPSNVVKVALSGISERNEIEELRKLGVNAFLIGSSLMRNPEKIKE
>RA46
MPRYLKGWLKDVVQLSLRRPSFRASRQRPIISLNERILEFNKRNITAIIAVYSRKSPSGLDVERDPIEYSKFMERYAVGLSIYTEEKYWNGSYE
TLRKIASSVSIPILMVDLIVKESQIDDAYNLGADTVVLIVSILTERELESLLEYARSYGMEPVIVINDENDLDIALRIGARFILIKSRDLETLE
INKENQRKLISMIPSNVVKVASWGISERNEIEELRKLGVNAFLIGSSLMRNPEKIKE
>RA45
MPRYLKGWLKDVVQLSLRRPSFRASRQRPIISLNERILEFNKRNITAIIALYSRKHPSGLDVERDPIEYSKFMERYAVGLSIWTEEKYVNGSYE
TLRKIASSVSIPILMVDFIVKESQIDDAYNLGADTVLLFVKILTERELESLLEYARSYGMEPLIVINDENDLDIALRIGARFIGIKSRDWETLE
INKENQRKLISMIPSNVVKVAMSGISERNEIEELRKLGVNAFLITYSLMRNPEKIKE
>RA42
MPRYLKGWLKDVVQLSLRRPSFRASRQRPIISLNERILEFNKRNITAIIALYSRKSPWGLDVERDPIEYSKFMERYAVGLVIATEEKYTNGSYE
TLRKIASSVSIPILMWDFIVKESQIDDAYNLGADTVLLIVKILTERELESLLEYARSYGMEPLIVINDENDLDIALRIGARFIKISSMDYETLE
INKENQRKLISMIPSNVVKVASSGISERNEIEELRKLGVNAFVIYSSLMRNPEKIKE
>RA6
MPRYLKGWLKDVVQLSLRRPSFRASRQRPIISLNERILEFNKRNITAIIAMYSRKSPWGLDVERDPIEYSKFMERYAVGLVILTEEKYANGSYE
TLRKIASSVSIPILMVDWIVKESQIDDAYNLGADTVVLVVKILTERELESLLEYARSYGMEPLIVINDENDLDIALRIGARFIKISSEDLETLE
INKENQRKLISMIPSNVVKVAAHGISERNEIEELRKLGVNAFLIGSSLMRNPEKIKE
>RA47
MPRYLKGWLKDVVQLSLRRPSFRASRQRPIISLNERILEFNKRNITAIIAGYMRKSPWGLDVERDPIEYSKFMERYAVGLAITTEEKYANGSYE
TLRKIASSVSIPILMADFIVKESQIDDAYNLGADTVALIVKILTERELESLLEYARSYGMEPLIKINDENDLDIALRIGARFIGIVSRDWETLE
INKENQRKLISMIPSNVVKVASYGISERNEIEELRKLGVNAFSIYSSLMRNPEKIKE
>RA39
MPRYLKGWLKDVVQLSLRRPSFRASRQRPIISLNERILEFNKRNITAIIAGYSRKSPTGLDVERDPIEYSKFMERYAVGLSILTEEKYFNGSYE
TLRKIASSVSIPILMTDFIVKESQIDDAYNLGADTVALIVKILTERELESLLEYARSYGMEPLIVITDENDLDIALRIGARFIKILSRDWETGE
INKENQRKLISMIPSNVVKVASSGISERNEIEELRKLGVNAFSIYSSLMRNPEKIKE
>RA36
MPRYLKGWLKDVVQLSLRRPSFRASRQRPIISLNERILEFNKRNITAIIAGYVRKGPWGLDVERDPIEYSKFMERYAVGLAIATEEKYWNGSYE
TLRKIASSVSIPILMTDFIVKESQIDDAYNLGADTVALIVKILTERELESLLEYARSYGMEPLIKINDENDLDIALRIGARFIGIVSADWETLE
INKENQRKLISMIPSNVVKVASFGISERNEIEELRKLGVNAFAIYSSLMRNPEKIKE
>RA35
MPRYLKGWLKDVVQLSLRRPSFRASRQRPIISLNERILEFNKRNITAIIAGYIRKSPSGLDVERDPIEYSKFMERYAVGLAITTEEKYGNGSYE
TLRKIASSVSIPILMADFIVKESQIDDAYNLGADTVALIVKILTERELESLLEYARSYGMEPLIKINDENDLDIALRIGARFIGIISRDWETLE
INKENQRKLISMIPSNVVKVASYGISERNEIEELRKLGVNAFSIYSSLMRNPEKIKE
>RA34
MPRYLKGWLKDVVQLSLRRPSFRASRQRPIISLNERILEFNKRNITAIIALYMRKSPWGLDVERDPIEYSKFMERYAVGLSITTEEKYANGSYE
TLRKIASSVSIPILMADFIVKESQIDDAYNLGADTVALIVKILTERELESLLEYARSYGMEPLIKINDENDLDIALRIGARFIGIVSRDWETLE
INKENQRKLISMIPSNVVKVASYGISERNEIEELRKLGVNAFSIGSSLMRNPEKIKE

V. Synthesis of S-4-hydroxy-4-(6-methoxynaphthalene-2-yl)butan-2-one (S-methodol)

The synthesis of S-methodol was performed in a stereo-selective manner using the method established by Zhou and Shan [5]. A 10 ml round bottom flask dried overnight in an oven was equipped with a magnetic stirrer, filled with D-proline (172.5 mg, 1.5 mmol, 30 mol-%) and (S)- 1,1'-Bi-2-naphthol (14.3 mg, 0.05 mmol, 1 mol-%) which were suspended in a mixture of acetone (p.A., 3 ml) and dimethyl sulfoxide (p.A., 1 ml) while stirring. After 15 min of stirring at 0°C, 6-methoxy-2-naphtaldehyde (931.1 mg, 5 mmol) was added to the flask; the flask was sealed and the suspension stirred vigorously for 48 h at 0°C. After completion of the reaction, 2 ml saturated ammonium chloride solution were added and the mixture stirred for 1 h. The mixture was extracted three times with 30 ml ethyl acetate, dried over magnesium sulphate and the solvent removed in a rotary evaporator. The resulting red oil was dissolved in 20 ml dichloromethane and adsorbed on SiO_2 followed by column chromatography on SiO_2 (d = 2.5 cm, l = 15 cm, cyclohexane/ethyl acetate 4:1) for elution of the aldehyde not turned over in the reaction. The reaction product was eluted with cyclohexane/ethyl acetate 2:3. After removal of the eluent in a rotary evaporator the product was a solid with light yellow colouring (211 mg, 904.7 µmol, 38% yield, 70% ee).

VI. References

[1] Krieger E, Darden T, Nabuurs SB, Finkelstein A, Vriend G. Making optimal use of empirical energy functions: force-field parameterization in crystal space. Proteins. 2004;57(4):678-83.

[2] Essmann U, Perera L, Berkowitz ML, Darden T, Lee H, Pedersen LG. A smooth particle mesh Ewald method. The Journal of Chemical Physics. 1995 Nov 15;103(19):8577-93.

[3] Krieger E, Nielsen JE, Spronk CA, Vriend G. Fast empirical pK a prediction by Ewald summation. Journal of Molecular Graphics and Modelling. 2006 Dec 31;25(4):481-6.

[4] Bjelic S, Kipnis Y, Wang L, Pianowski Z, Vorobiev S, Su M, et al. Exploration of alternate catalytic mechanisms and optimization strategies for retroaldolase design. Journal of Molecular Biology. 2014;426(1):256-71.

[5] Zhou Y, Shan Z. Chiral diols: A new class of additives for direct aldol reaction catalyzed by L-proline. The Journal of Organic Chemistry. 2006;71(25): 9510-9512.
