# Supplementary figures and images for: Rosetta:MSF: a modular framework for multi-state computational protein design
Source: PLoS Comput Biol. 2017 Jun 12;13(6):e1005600. doi: 10.1371/journal.pcbi.1005600 (PMC5484525; doi:10.1371/journal.pcbi.1005600)

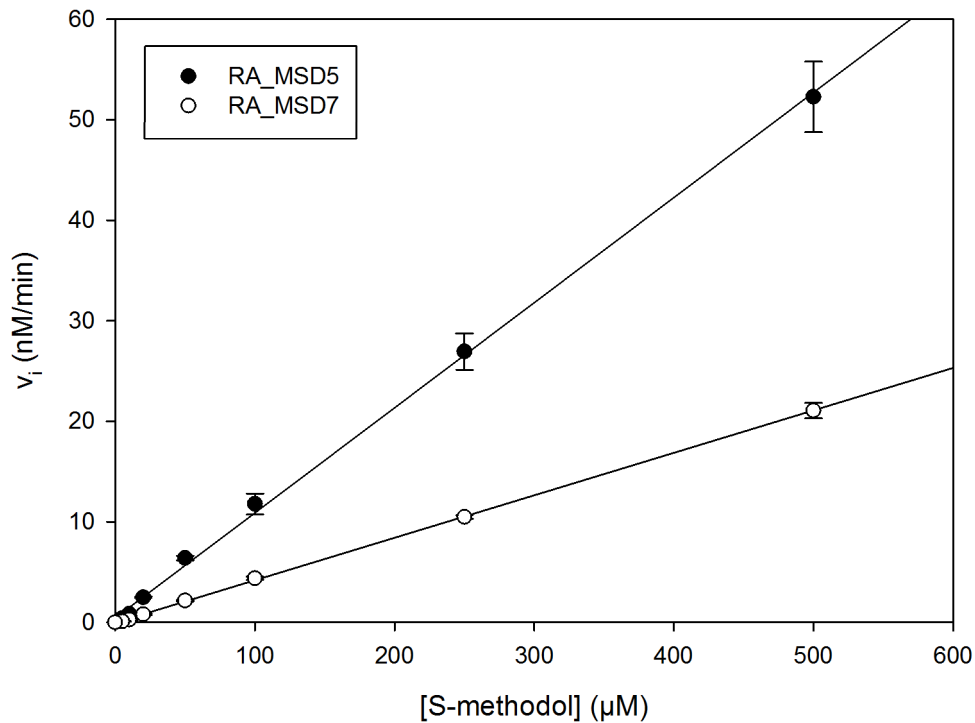

Supplement: S1 Fig — Due to the low affinity of the two designs for S-methodol, only the linear part of the substrate saturation curves could be determined. The slopes yielded catalytic efficiencies (kcat/KM) of 3.47 × 10^−2^ and 1.41 × 10^−2^ M-1s-1 for RA_MSD5 and RA_MSD7, respectively. (PDF) [file pcbi.1005600.s004.pdf]
